# Operando Spectroscopy Unveils the Catalytic Role of Different Palladium Oxidation States in CO Oxidation on Pd/CeO_2_ Catalysts

**DOI:** 10.1002/anie.202200434

**Published:** 2022-04-05

**Authors:** Valery Muravev, Jérôme F. M. Simons, Alexander Parastaev, Marcel A. Verheijen, Job J. C. Struijs, Nikolay Kosinov, Emiel J. M. Hensen

**Affiliations:** ^1^ Laboratory of Inorganic Materials and Catalysis Department of Chemical Engineering and Chemistry Eindhoven University of Technology P.O. Box 513 5600 MB Eindhoven The Netherlands; ^2^ Department of Applied Physics Eindhoven University of Technology P.O. Box 513 5600 MB Eindhoven The Netherlands; ^3^ Eurofins Material Science Netherlands BV 5656AE Eindhoven The Netherlands

**Keywords:** CO Oxidation, Ceria, NAP-XPS, Operando DRIFTS, Structure–Activity Relationships

## Abstract

Aiming at knowledge‐driven design of novel metal–ceria catalysts for automotive exhaust abatement, current efforts mostly pertain to the synthesis and understanding of well‐defined systems. In contrast, technical catalysts are often heterogeneous in their metal speciation. Here, we unveiled rich structural dynamics of a conventional impregnated Pd/CeO_2_ catalyst during CO oxidation. In situ X‐ray photoelectron spectroscopy and operando X‐ray absorption spectroscopy revealed the presence of metallic and oxidic Pd states during the reaction. Using transient operando infrared spectroscopy, we probed the nature and reactivity of the surface intermediates involved in CO oxidation. We found that while low‐temperature activity is associated with sub‐oxidized and interfacial Pd sites, the reaction at elevated temperatures involves metallic Pd. These results highlight the utility of the multi‐technique operando approach for establishing structure–activity relationships of technical catalysts.

## Graphical abstract

Establishing structure–activity relationships for practical catalysts requires a multi‐technique operando approach. Using advanced spectroscopic tools and kinetic methods the catalytic role of different Pd oxidation states in CO oxidation was unveiled for Pd/CeO_2_ catalysts. Single‐atom Pd–oxo species and Pd−O−Ce interface are responsible for low‐temperature activity, whereas metallic Pd sites significantly contribute only at elevated temperature.

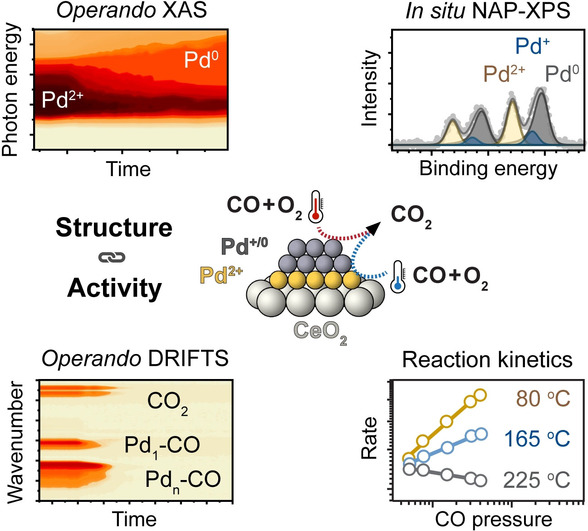

## Introduction

In search of guiding principles for catalyst design, identifying structure–performance relationships has become a main paradigm of modern catalysis research.[^1^, ^2^, ^3^] CO oxidation has been long used to probe the reactivity of metals.[^4^, ^5^, ^6^, ^7^, ^8^] Noble metal nanoparticles are typical catalysts for CO oxidation used in automotive exhaust neutralizers,^[9]^ gas sensors,^[10]^ and fuel cells.^[11]^ When deposited on inert supports such as alumina, silica or zirconia, noble metals suffer from CO poisoning, which limits their low‐temperature performance.[^12^, ^13^, ^14^] Reducible oxide supports and especially ceria can be used to overcome this problem owing to specific catalytic sites at the metal–support interface.[^15^, ^16^, ^17^, ^18^]

Cargnello et al. showed that nanoparticles of Pt, Pd and Ni deposited on ceria are active in low‐temperature CO oxidation (<150 °C)^[19]^ due to catalytic sites at the metal–ceria interface. Maximizing the contribution of interfacial sites can increase the overall activity of the catalyst and, at the same time, improve the utilization degree of often expensive transition metals. For instance, a number of works reported that ceria‐supported Pd catalysts with a high metal dispersion display excellent CO oxidation activity.[^20^, ^21^, ^22^, ^23^] Synthesis of well‐defined Pd/CeO_2_ single‐atom catalysts has only recently been developed.[^24^, ^25^, ^26^, ^27^] The high reactivity of these materials for low‐temperature CO oxidation has been linked to the oxidized nature of isolated Pd atoms.[^24^, ^25^, ^26^] In the presence of other reactants (i.e., in model exhaust gas feeds), however, few‐atom Pd clusters deposited on CeO_2_(−Al_2_O_3_) nanocomposites exhibited notably higher CO oxidation activity than single‐atom catalysts.^[28]^

Structure–activity relations obtained for metal‐ceria systems with well‐defined metal speciation aid the design of practical CO oxidation catalysts based on Pd/CeO_2_. Nonetheless, typical metal‐oxide catalysts prepared by conventional preparation methods (e.g., impregnation or precipitation) often lack uniformity and contain a mixture of metal nanoparticles, clusters, and single atoms already in the as‐prepared state.[^7^, ^29^, ^30^, ^31^] Moreover, these species can transform into each other depending on the reaction conditions.[^32^, ^33^, ^34^, ^35^] For Pd/CeO_2_, several works demonstrated a coexistence and, in fact, synergy between PdO_*x*_ clusters and Pd atoms in the CeO_2_ lattice for the oxidation of CO at low temperature.[^36^, ^37^, ^38^] Other reports stressed the importance of metallic Pd sites for optimal catalytic performance.[^39^, ^40^] Despite all these efforts, the lack of operando spectroscopy data where structure and activity of Pd/CeO_2_ are determined simultaneously hampers the understanding of the catalytic roles of different Pd species. Moreover, it remains unclear whether activity descriptors derived for well‐defined nanoparticles or single atoms of Pd supported on ceria are applicable for catalysts prepared by conventional methods.

Here, a Pd/CeO_2_ catalyst prepared by wet impregnation of a commercially available ceria support was studied by a set of complementary operando and in situ spectroscopy tools. We used operando X‐ray absorption spectroscopy (XAS) to interrogate the changes in Pd speciation as a function of time, temperature, and composition of the reaction mixture. Using in situ near‐ambient pressure X‐ray photoelectron spectroscopy (NAP‐XPS), we identified various Pd species during CO oxidation with high surface and chemical sensitivity. We applied time‐resolved operando diffuse reflectance infrared Fourier transform spectroscopy (DRIFTS) to follow the evolution of adsorbates on Pd surface and determine the nature of active and spectator species in low‐temperature CO oxidation. Finally, correlation of the spectroscopic data with results of steady‐state and transient reaction kinetic experiments completed a mechanistic picture of the dynamic role of different Pd states in CO oxidation.

## Results and Discussion

### Structure and CO Oxidation Activity of Pd/CeO_2_ Catalyst

**Figure 1:**
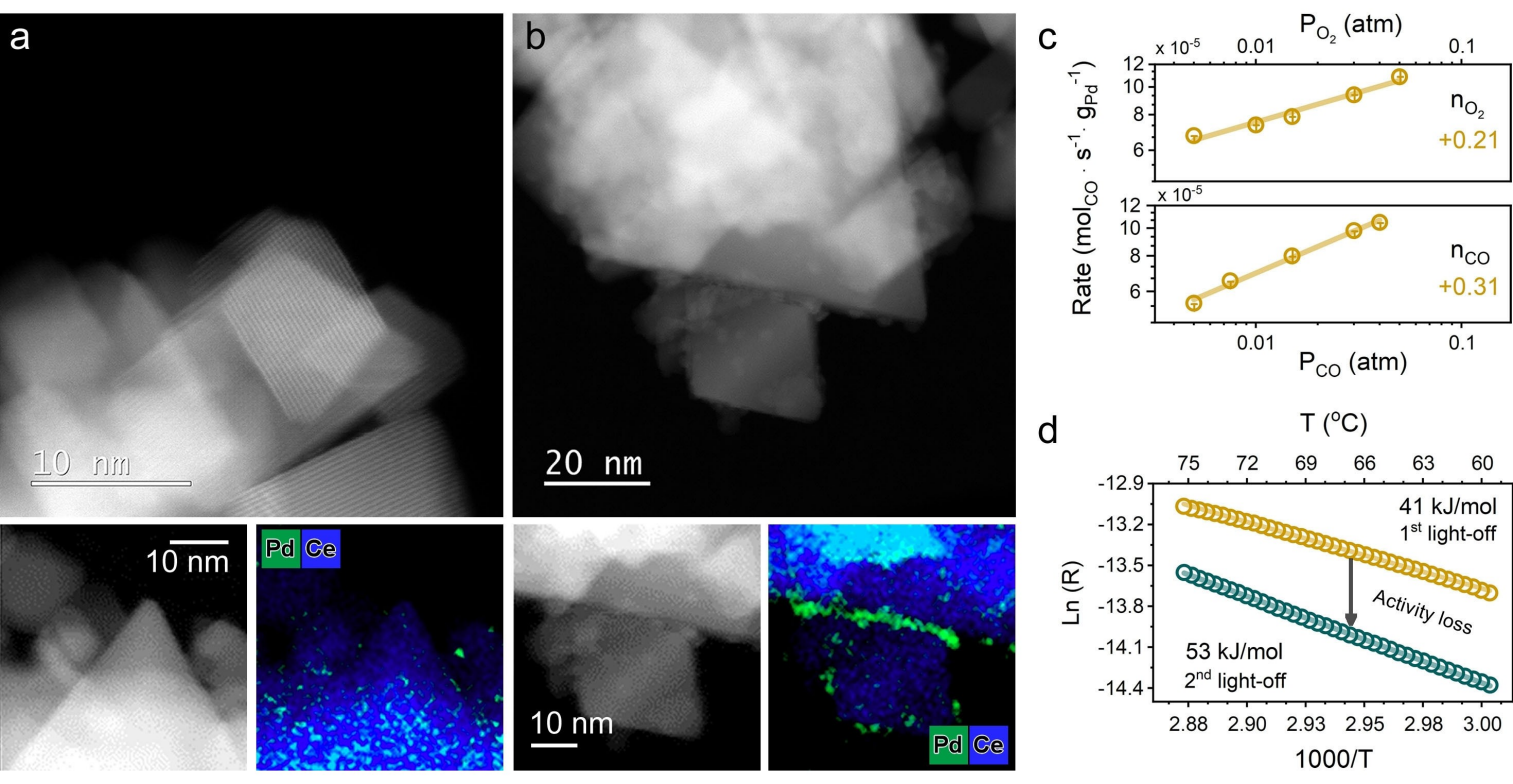
Structure and activity of Pd/CeO_2_ catalyst. HAADF‐STEM and EDX mapping of fresh Pd/CeO_2_ catalyst: a) region with highly‐dispersed Pd species; b) region with small clusters and nanoparticles of Pd oxide. c) Steady‐state catalytic activity of the Pd/CeO_2_ in CO oxidation as a function of pressure of reactants at 80 °C. GHSV≈240 000 mL g_cat_^−1^ h^−1^_,_ conversion kept below 5 %. Error bars represent five consequent measurements. d) Arrhenius plot for low‐temperature catalytic activity of Pd/CeO_2_ in CO oxidation. Yellow circles represent the first light‐off run (RT to 300 °C), cyan circles stand for the second run. Reaction conditions: 1 % CO and 1 % O_2_ in He (GHSV≈180 000 mL g_cat_^−1^ h^−1^), conversion kept below 10 %.

A commercial ceria support was loaded with 1 wt % Pd by wet impregnation using palladium nitrate as a precursor (sample denoted as Pd/CeO_2_). No Pd or PdO phases were detected by powder X‐ray diffraction and Raman spectroscopy in the as‐prepared state of Pd/CeO_2_ (Figure S1). As shown in Figure 1a, b, high‐resolution high‐angle annular dark‐field scanning transmission electron microscopy (HAADF‐STEM) together with energy‐dispersive X‐ray spectroscopy (EDX) mapping revealed a non‐uniform speciation of Pd. While part of Pd is highly dispersed (Figure 1a, Figure S2), another part is present as nanoparticles of ≈3 nm (Figure 1b, Figure S3). Ex situ X‐ray absorption near‐edge structure (XANES) analysis demonstrated that Pd is in the oxidic state in the as‐prepared sample (Figure S4). Fitting of extended X‐ray absorption fine structure (EXAFS) data confirmed the high dispersion of Pd (CN_Pd−Pd_≈4, vs. 12 in PdO_bulk_, Table S1). Thus, Pd–oxo species in the as‐prepared catalyst vary in size from few‐atom clusters to 3 nm nanoparticles. The steady‐state CO oxidation activity was evaluated at 80 °C. The reaction orders with respect to CO and O_2_ were +0.31 and +0.21, respectively (Figure 1c). The positive reaction order in CO points to the absence of CO poisoning at low temperature, which has earlier been associated with CO oxidation taking place at the Pd−CeO_2_ interface.[^18^, ^19^, ^24^] Previously, we reported that, unlike reduced metal nanoparticles, single Pd atoms on CeO_2_ exhibited a positive order in CO, while the reaction order in O_2_ was negative.^[24]^ The positive reaction order in O_2_ observed in the present work might therefore indicate that the fraction of single‐atom Pd species in impregnated Pd/CeO_2_ catalyst is low under reaction conditions. This is consistent with the lower activity of Pd/CeO_2_ in low‐temperature CO oxidation (Figure S5) in comparison to a single‐atom Pd_1_/CeO_2_ catalyst.^[24]^ Repeated CO oxidation light‐off cycles over the same catalyst revealed that the activity at low temperature (<100 °C) decreased during the second light‐off run (Figure 1d). Together with an increase in apparent activation energies (41 vs. 53 kJ mol^−1^), these results point to structural changes in the catalyst during CO oxidation. Accordingly, we employed operando X‐ray absorption spectroscopy (XAS) at the Pd K‐edge to follow the structure of Pd/CeO_2_ during CO oxidation.

### Structural Dynamics of Pd/CeO_2_ Catalyst during Low‐Temperature CO Oxidation

**Figure 2:**
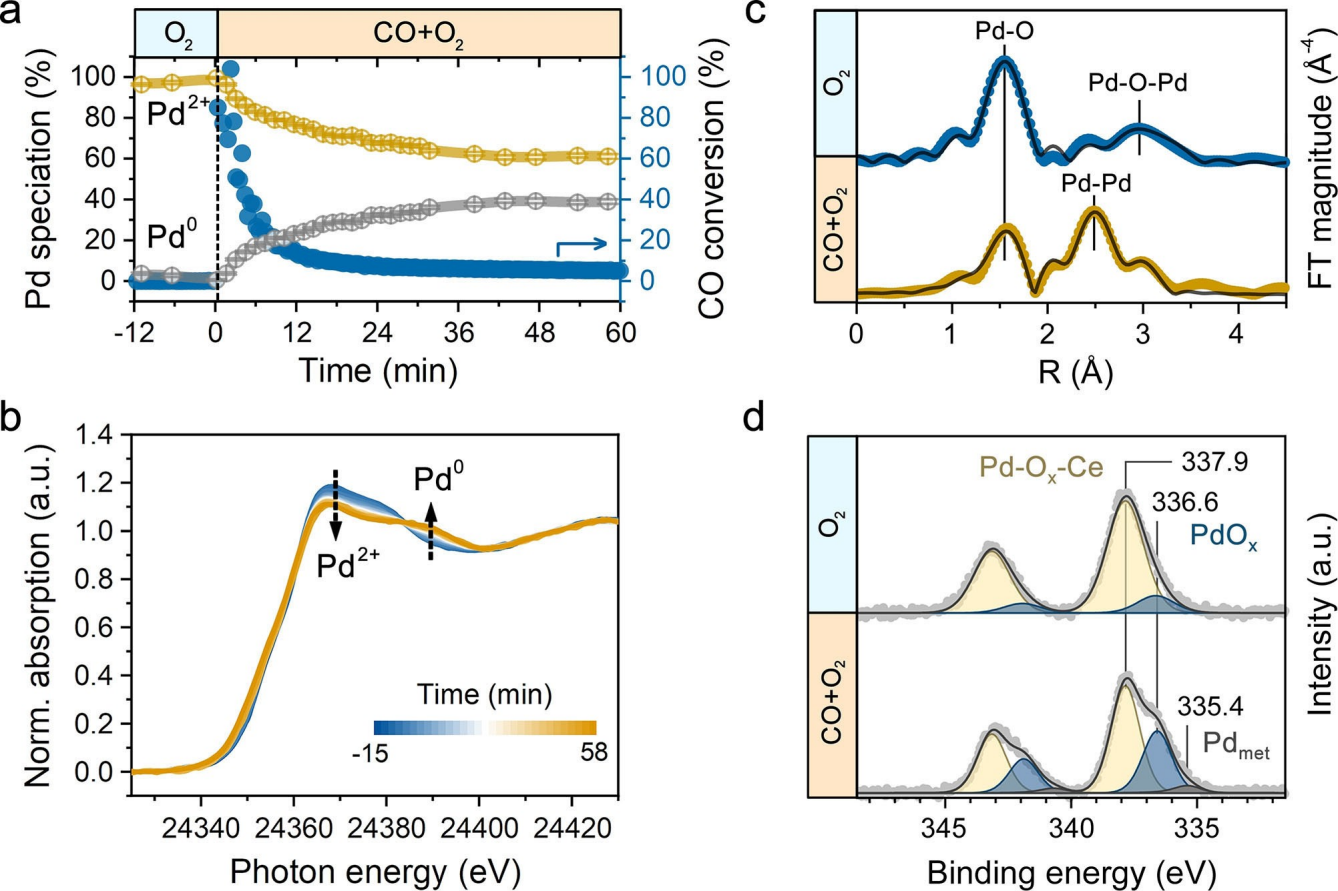
Reaction driven structural dynamics of Pd/CeO_2_ catalyst. a) Evolution of Pd oxidation state (derived from LCF analysis) and CO conversion during operando XAS experiment. b) Operando XAS at Pd K‐edge during a switch from O_2_ to CO+O_2_ at 80 °C. XAS data acquired in the middle of the catalyst bed. Conditions: “O_2_”—20 % O_2_ in He; “CO+O_2_”—1 % CO and 1 % O_2_ in He, total flow 100 mL min^−1^. c) R‐space plot of EXAFS data for the sample before the switch (blue) and after 60 min stabilization in the reaction mixture at 80 °C (amber). d) In situ NAP‐XPS of Pd 3d before and after the switch from O_2_ to CO+O_2_ (1 : 1) at 80 °C (2 mbar). Each NAP‐XPS measurement was performed for ≈2.5 h after complete replacement of the gas phase in the NAP‐cell.

Taking advantage of the high penetrating power of X‐rays at the Pd K‐edge (≈24.4 keV), we could use a conventional quartz plug‐flow reactor and high space velocities (≈200 000 mL g_cat_^−1^ h^−1^) for XAS measurements. Complemented by online mass spectrometry (MS), this setup allows operando characterization of Pd in Pd/CeO_2_ catalyst during CO oxidation. In the experiment the catalyst was pretreated in oxygen at 300 °C followed by cooling to 80 °C. Then the oxygen feed was switched to the reaction mixture—1 vol % CO and 1 % vol% O_2_ in He. XANES analysis revealed substantial spectral changes upon a switch from O_2_ to CO+O_2_ (Figure 2a). Using spectra of O_2_‐pretreated and reduced catalyst as standards for Pd^2+^ and Pd^0^ species (Note S1) for linear combination fitting (LCF), we found that the Pd oxide species initially present in Pd/CeO_2_ catalyst were reduced (Figure 2b). Within 30 min, around 40 % of Pd^2+^ was transformed into Pd^0/δ+^ species. These changes in the Pd oxidation state are accompanied by a substantial decrease in the CO oxidation activity. While initially the CO conversion was around 90 % (Note S2), the conversion decreased to ≈5 % after 40 min. These results indicate that highly dispersed Pd–oxo species, present in the fresh catalyst, are highly active in low‐temperature CO oxidation but not stable. In situ EXAFS data reveal that the second part of deactivation process (>30 min) goes together with the appearance of a Pd−Pd shell due to metallic Pd (Figure 2c and Figure S7). The coordination numbers for Pd−Pd scattering paths for the Pd and Pd‐oxide species were below 6, suggesting that metallic/sub‐oxidized Pd nanoparticles are formed under low‐temperature CO oxidation conditions.

As EXAFS is a bulk averaging technique, we cannot distinguish a mixture of Pd‐oxide and Pd metal from sub‐oxide Pd phase that can be present on the surface under reaction conditions.^[41]^ Thus, we studied the surface speciation of Pd by in situ NAP‐XPS. After pretreatment in O_2_, the Pd 3d spectrum (Figure 2d) contains two principal components: i) ≈87 % of Pd 3d_5/2_ at 337.9 eV due to highly dispersed Pd^2+^ in strong interaction with ceria (Pd−O_*x*_−Ce) and ii) ≈13 % Pd 3d_5/2_ at 336.6 eV related to sub‐oxidized PdO_*x*_ species.[^20^, ^24^, ^42^] This shows that both highly dispersed and clustered Pd–oxo species are present in the pretreated catalyst. When the catalyst was exposed to the CO+O_2_ reaction mixture in the NAP cell, partial reduction of Pd^2+^ took place resulting in ≈35 % of sub‐oxidized and ≈5 % of metallic species. Owing to the high sensitivity of the 3d core‐level XPS to intermediate charge states, we can conclude that at low reaction temperature most of Pd^2+^ reduced to sub‐oxidized Pd^δ+^ state instead of metallic Pd^0^. The absence of severe Pd sintering under low‐temperature CO oxidation conditions is confirmed by similar values of atomic Pd/Ce ratios determined by XPS (≈0.04) in both O_2_ and CO+O_2_ atmospheres.

### Probing the Adsorbate Bonding at Pd Sites by Time‐Resolved Operando DRIFTS

**Figure 3:**
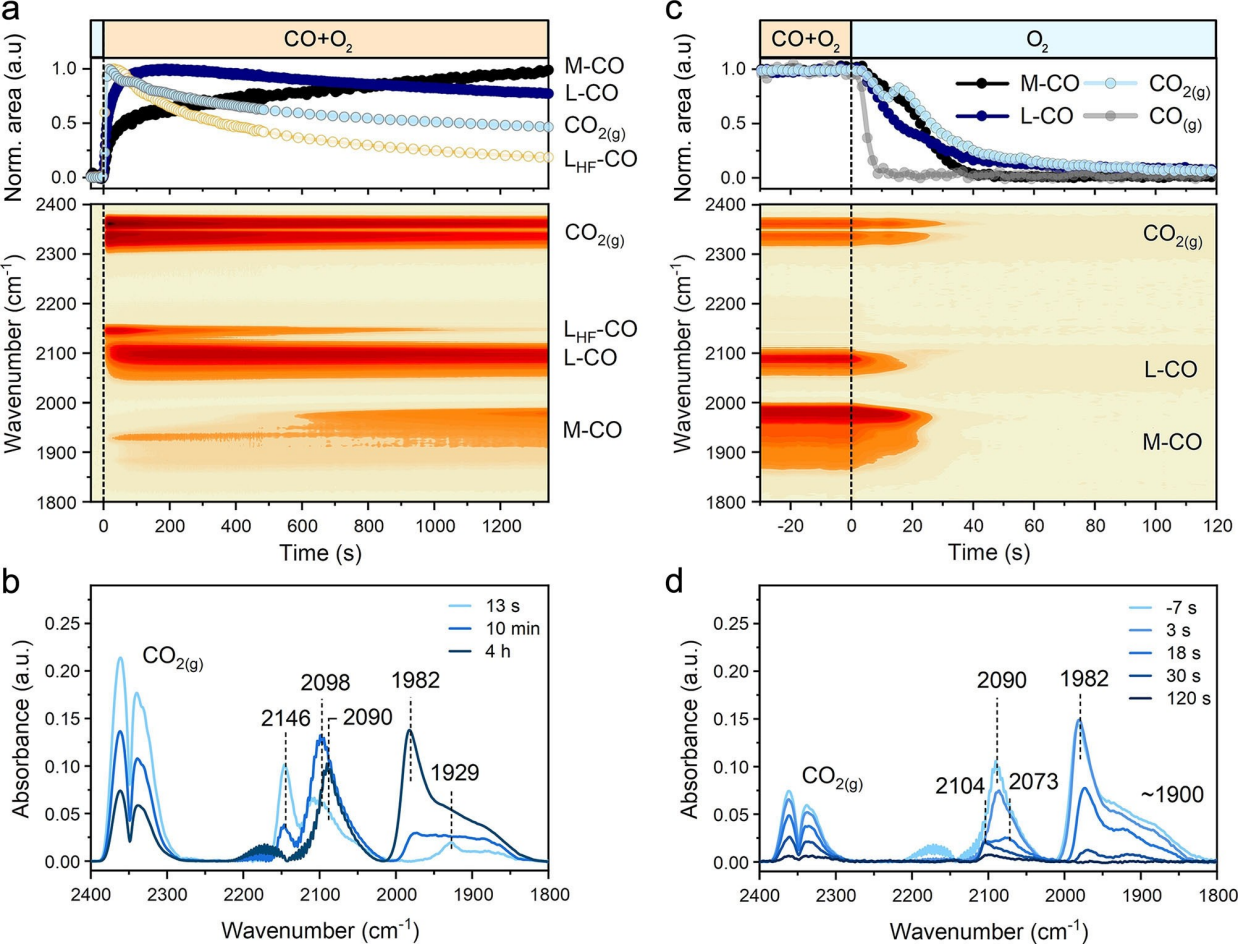
Response of Pd carbonyl species to chemical transients monitored by time‐resolved DRIFTS. a) DRIFTS data acquired during a forward switch from O_2_ to CO+O_2_ at 80 °C. b) Selected FTIR spectra acquired during a forward switch from O_2_ to CO+O_2_ at 80 °C. c) DRIFTS data acquired during a backward switch from CO+O_2_ to O_2_ at 80 °C. d) Selected FTIR spectra acquired during a backward switch from CO+O_2_ to O_2_ at 80 °C. Conditions: “O_2_”—1 % O_2_ in He; “CO+O_2_”—1 % CO+1 % O_2_ in He; “CO”—1 % CO in He. Total flow 100 mL min^−1^.

Insights into the bonding and reactivity of CO molecules adsorbed on the surface of Pd were obtained from chemical transient kinetic experiments monitored by operando DRIFTS with high temporal resolution of 0.6 s^−1^. Figure 3a shows the changes in the carbonyl absorption bands upon a switch from O_2_ to CO+O_2_ at 80 °C. We found that CO band at 2146 cm^−1^ was the dominant one during the first seconds of the reaction (Figure 3a, b). This high‐frequency band (denoted here as L_HF_−CO) has been earlier assigned to linear carbonyls of single‐atom Pd species.[^24^, ^25^, ^26^] These atomically dispersed oxidized Pd species are highly reactive but require a nanostructured ceria support with enhanced oxygen mobility to maintain their isolated nature during CO oxidation.^[24]^ Clearly, on the surface of the relatively large ceria particles used in the present study the oxidized Pd single atoms are prone to reduction and clustering. The decrease in L_HF_−CO goes together with the appearance of additional bands at ≈2090 cm^−1^ and ≈1980–1850 cm^−1^ due to linear (L−CO) and multi‐bonded (M−CO) carbonyls of sub‐oxidized and metallic Pd, respectively.[^24^, ^25^, ^26^] The reduction and partial sintering of Pd‐oxo species is accompanied by a significant decrease of the CO oxidation activity, as follows from the decrease of the IR band intensity of gas‐phase CO_2_ in the DRIFTS cell and the decrease of the *m*/*z*=44 signal in MS (Figure S8). These changes are consistent with the results of operando XAS characterization, indicating that the initial high catalytic activity is linked to highly dispersed Pd‐oxo species.

**Figure 4:**
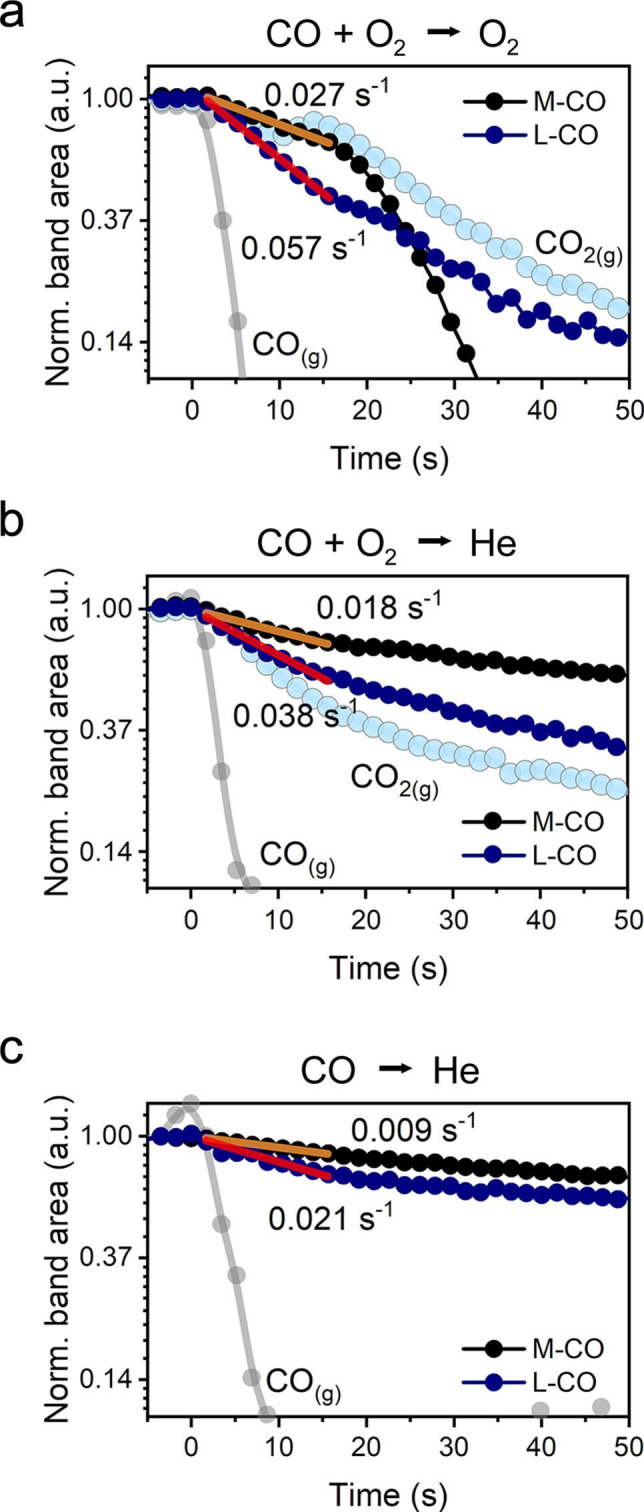
Kinetics of the removal of Pd carbonyls during chemical transient experiments. Semi‐logarithmic plots of FTIR band areas as a function of time for: a) CO+O_2_ to O_2_ transient; b) CO+O_2_ to He transient; c) CO to He transient. Conditions: 80 °C; “O_2_”—1 % O_2_ in He; “CO+O_2_”—1 % CO+1 % O_2_ in He; “CO”—1 % CO in He. Total flow 100 mL min^−1^.

After steady state CO oxidation activity was reached and no further changes in IR spectra of the operating catalyst were seen, the backward transient from CO+O_2_ to O_2_ was performed. As shown in Figure 3c, d, CO is completely removed from the gas phase in about 8 s following the switch. In contrast, the removal of adsorbed CO is substantially slower, and the responses of the L−CO and M−CO carbonyls exhibited very different kinetics (Figure 3c). By using rapid‐scan FTIR, we could resolve two stages of carbonyl evolution during the switch from CO+O_2_ to O_2_. The first one (*t*<17 s) was found to be linear in the semi‐logarithmic coordinates (Figure 4a) and gave rise to rate constants for first‐order L−CO and M−CO decomposition of ≈0.057 s^−1^ and ≈0.027 s^−1^, respectively. Thus, the initial rate of removal of linearly adsorbed CO on sub‐oxidized Pd sites is nearly twice faster than the removal of multi‐bonded CO on metallic Pd. However, in the second stage (*t*>17 s), M−CO decomposed much faster (*k*≈0.145 s^−1^) than L−CO (*k*≈0.037 s^−1^). When M−CO started to decay rapidly (at *t*≈20 s), the decrease in L−CO band area slowed down (Figure S9), likely due to the interconversion between multi‐bonded and linearly adsorbed CO species.[^43^, ^44^]

The two‐step nature of the carbonyl decomposition can be explained by differences in the CO oxidation mechanism at high and low coverages of CO. We hypothesize that during the first stage of CO+O_2_ to O_2_ transient, CO oxidation proceeds mainly at the Pd−CeO_2_ interface, because metallic sites remain poisoned due to the high CO coverage. In the second phase of the transient, the CO coverage decreases, allowing oxygen to adsorb on metallic Pd where oxidation of CO can take place. CO oxidation involving facile oxygen dissociation on bridge and/or hollow sites of metallic Pd surface,^[45]^ can explain the fast decay of M−CO and the peak in CO_2_ gas phase evolution at ≈17 s after the switch (Figure 3c and Figure S10).

To understand the effect of CO desorption on the observed transient responses, we also performed a transient experiment in which the CO+O_2_ reaction mixture was replaced by inert He (Figure S11). The disappearance of Pd carbonyls proceeded much slower (≈200 s) than upon the CO+O_2_ to O_2_ switch (≈60 s) and with much lower rate constants (Figure 4b) for the decays of L−CO (≈0.038 s^−1^) and M−CO (≈0.018 s^−1^) than for the backward O_2_ transient (Figure 4a). We attribute this difference to oxidation of adsorbed CO by O_2_ in the CO+O_2_ to O_2_ transient. Recent works suggest that oxidation of chemisorbed CO at low temperature can also occur in inert atmosphere due to the supply of oxygen atoms from ceria to interface sites.[^17^, ^46^] To deplete reactive oxygen atoms, the catalyst initially operated at steady state in a CO+O_2_ mixture at 80 °C was exposed to a feed of 1 % CO in He. As a result of surface reduction, we observed a bathochromic shift (≈7 cm^−1^) of both L−CO and M−CO bands and an increased intensity of multi‐bonded carbonyls (Figure S12). The total band area of Pd carbonyls for the catalyst exposed to CO is not very different from the CO+O_2_ case, indicating that the CO coverage is high during steady‐state CO oxidation. This is consistent with the mechanistic proposal that low‐temperature CO oxidation at high CO coverages occurs at the Pd−CeO_2_ interface, while the rest of Pd surface is poisoned by CO. When the intensity of the Pd carbonyl bands stabilized after exposure of the catalyst to CO, a switch from CO to He feed was performed. In this case, the removal of carbonyls took much longer (≈600 s) than for the switches from CO+O_2_ to O_2_ (≈60 s) and CO+O_2_ to He (≈200 s). The rate constants for decomposition of L−CO (≈0.021 s^−1^) and M−CO (≈0.009 s^−1^) in He after CO exposure (Figure 4c) were much lower than upon the CO+O_2_ to He switch. This difference can be attributed to stronger adsorption of CO on more reduced Pd sites and the depletion of reactive oxygen atoms at the Pd−CeO_2_ interface.

To investigate the latter aspect, we performed an identical set of experiments on a reference sample in which lattice oxygen mobility and its impact on CO oxidation kinetics can be neglected. For this purpose, non‐reducible ZrO_2_ support^[47]^ was impregnated with 1 wt % Pd (sample denoted as Pd/ZrO_2_). As shown in Figure S13, this catalyst was much less active than Pd/CeO_2_ in low‐temperature CO oxidation and followed a conventional Langmuir–Hinshelwood mechanism.[^48^, ^49^] The presence of similar L−CO and M−CO bands under identical reaction conditions (Figure S14) facilitates a comparison of the kinetics of Pd carbonyl removal from Pd/CeO_2_ and Pd/ZrO_2_. During the switch from CO+O_2_ to O_2_ over Pd/ZrO_2_, carbonyl removal was much slower (≈600 s) than for Pd/CeO_2_. This difference can be explained by the absence of reactive lattice oxygen in ZrO_2_. Strikingly, the kinetics of the disappearance of L−CO and M−CO bands for Pd/ZrO_2_ during CO+O_2_ to He (Figure S15) and CO to He (Figure S16) switches were similar. From this, we infer that reactive ceria lattice oxygen is the key to efficient low‐temperature CO oxidation on supported Pd.

### Structural Evolution of Pd during CO Oxidation at Elevated Temperature

**Figure 5:**
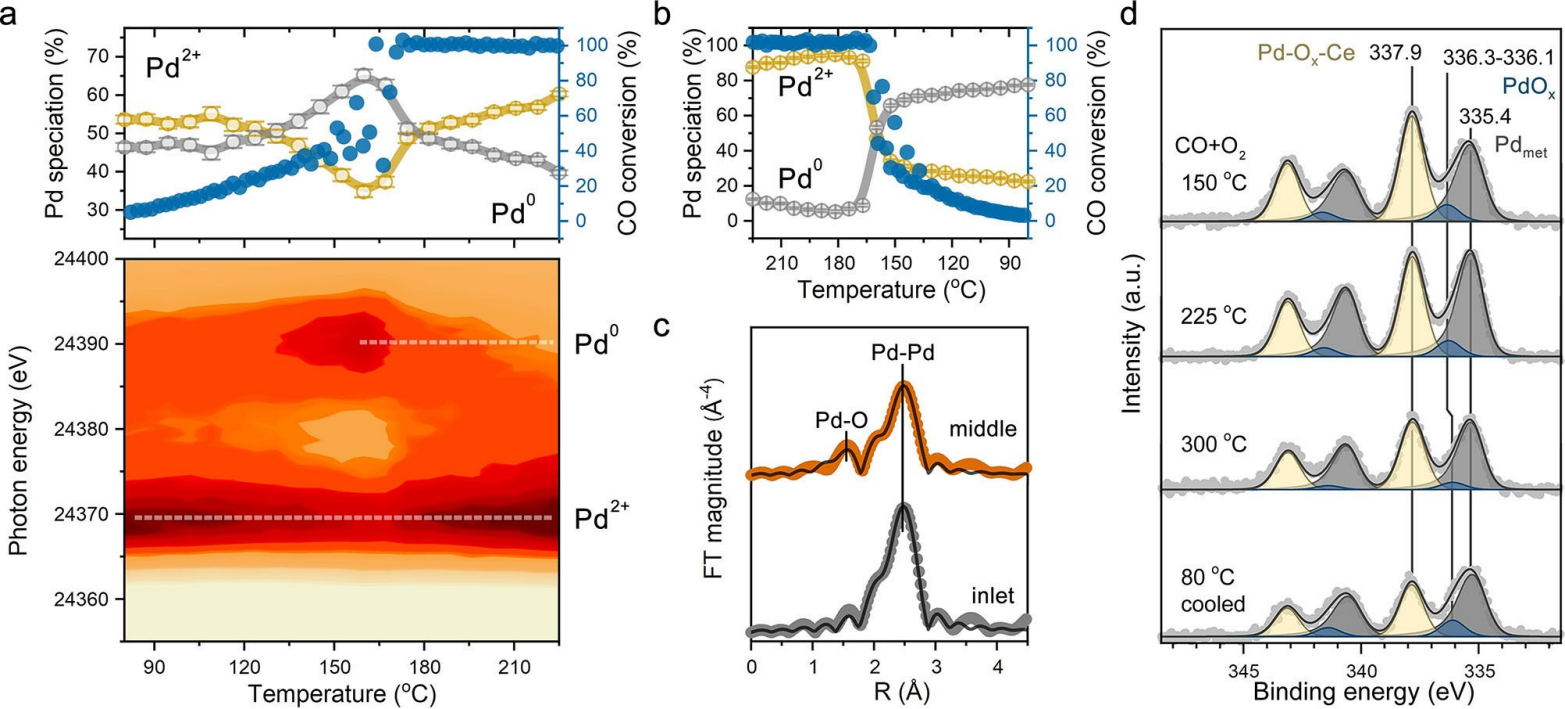
Evolution of Pd during CO oxidation as a function of temperature. a) Operando XAS at Pd K‐edge during heating in 1 % CO and 1 % O_2_ in He (total flow 100 mL min^−1^). XAS data acquired close to the middle of the catalyst bed. b) Evolution of Pd oxidation state upon cooling in reaction mixture after light‐off test. c) R‐space EXAFS data for different parts of the catalyst bed cooled to 80 °C after the reaction. d) In situ NAP‐XPS of Pd 3d for the catalyst exposed to CO+O_2_ (1 : 1) mixture (2 mbar).

Figure 5a shows that heating of Pd/CeO_2_ in reaction mixture up to ≈150 °C led to increase in CO oxidation activity and reduction of Pd–oxo species. At around 160 °C the ≈50 % CO conversion was reached and reduction of Pd–oxo species stopped. Further heating led to reoxidation of Pd^0/δ+^ to Pd^2+^. Oscillations in the catalytic activity caused by the exothermicity of the reaction (see Note S2) were not observed after reaching full CO conversion (≈170 °C). After stabilization for 30 min at 225 °C, around 80 % of Pd was oxidized. Reoxidation of Pd after ignition of CO oxidation has been observed before for Pd on redox‐inactive alumina, suggesting that this phenomenon is related to the chemical properties of Pd−PdO clusters rather than to the ceria support specifically.[^50^, ^51^]

**Figure 6:**
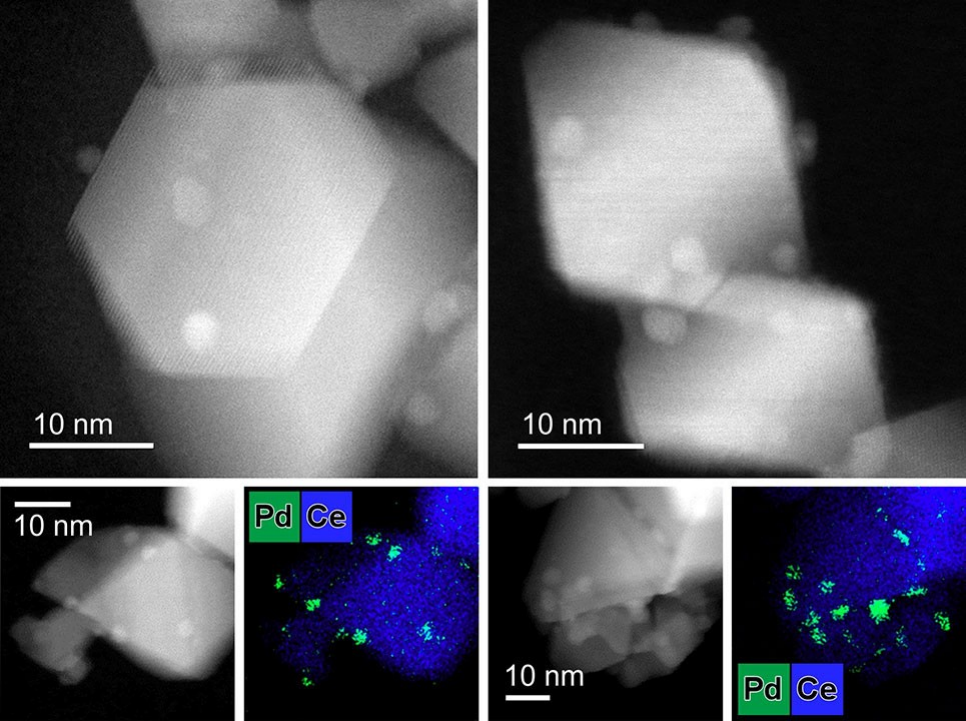
HAADF‐STEM and EDX mapping of the Pd/CeO_2_ catalyst after CO oxidation. In contrast to as‐prepared state, Pd is predominantly present as ≈3 nm Pd/PdO nanoparticles after CO oxidation at elevated temperature.

Upon cooling (Figure 5b), reduction of Pd started at ≈165 °C, resulting in around 25 % of Pd^2+^ at 80 °C. These light‐off experiments show that the catalyst did not return to the initial state preceding ignition (≈55 % of Pd^2+^). EXAFS analysis revealed a decrease in the Pd−O coordination number to ≈2 and an increase in Pd−Pd coordination number to 7 (Figure 5c, top) from respective initial values of ≈4 and ≈5 for the fresh catalyst (Figure 2c, top). TEM images and EDX mapping of the used catalyst showed that Pd was present as ≈3 nm particles (Figure 6 and Figure S18). The reduction and sintering of highly dispersed Pd‐oxo species during CO oxidation at elevated temperature can explain the partial loss of low‐temperature catalytic performance during the repeated light‐off tests (Figure 1d).

The temperature and concentration gradients along the catalyst bed that occur at high temperatures and/or high conversion levels can significantly contribute to heterogeneity in the Pd speciation.[^15^, ^51^, ^52^] After the CO oxidation light‐off test on Pd/CeO_2_, XAS was performed close to the feed side (2 mm), in the middle (6 mm, standard position) and close to the exit (14 mm) of the catalyst bed (15 mm total). XANES indicated a significant difference in Pd state at the inlet position (Figure S19) as compared to the middle and the end of the bed, which appeared to be rather similar. LCF analysis revealed an almost two times lower concentration of Pd^2+^ in the beginning of the catalyst bed with respect to the other segments. Fitting of the EXAFS data (Figure 5c and Table S1) acquired in this position reveals a more severe reduction and sintering of Pd species. This result indicates that at elevated temperature and full conversion of CO only the front part of the catalyst bed is used to convert the feed mixture, while the rest of the catalyst is only exposed to a mixture of O_2_ and CO_2_. This also explains a substantial degree of Pd reoxidation in the middle of the reactor observed at *T*>170 °C. Accordingly, we can explain the observed reduction of Pd upon cooling from 225 °C to 80 °C (Figure 5b). When the temperature decreases and CO is not fully converted anymore, the middle of the bed starts to be exposed to CO, which leads to Pd reduction.

To disentangle the reaction‐driven changes in Pd speciation from the effects of local temperature and concentration gradients along the catalyst bed, we performed an in situ NAP‐XPS study. Upon heating the catalyst from 80 °C to 150 °C in the reaction mixture, a considerable fraction of oxidized Pd−O_*x*_−Ce and PdO_*x*_ species transformed into metallic Pd (Figure 5d), in line with operando XAS data showing reduction of Pd (Figure 5a). Further heating to 225 °C resulted in a slightly higher fraction of Pd metal (≈53 %), whereas nearly full reoxidation of Pd was observed during the operando XAS experiment in a catalytic bed. Even at 300 °C, no notable reoxidation of Pd was observed by NAP‐XPS. This can be explained by the incomplete CO conversion in the NAP cell (Figure S20), where the gases flow over the catalyst surface instead of passing through the catalyst bed as in operando XAS tests. The in situ determined Pd/Ce surface atomic ratio decreased at elevated temperatures (Table S2), pointing to sintering of metallic Pd. When the catalyst was cooled to 80 °C in the reaction mixture, only a slight decrease in the Pd^2+^ fraction (≈9 %) was seen by NAP‐XPS. This is also in contrast to the notable reduction observed in the corresponding operando XAS measurements, which is due to the exposure of the whole catalyst bed to CO at incomplete conversion. Despite these differences, the average oxidation state of Pd in the used catalyst after the first light‐off cycle determined by XPS and XAS were similar (Pd^δ+^, *δ*≈0.4–0.6), indicating the minor effect of the pressure gap between the two techniques (2 mbar in NAP‐XPS and 20 mbar in XAS).

Altogether we conclude that substantial structural changes in Pd/CeO_2_ take place during CO oxidation light‐off. Small sub‐oxidized Pd clusters formed at low temperature undergo partial reduction and sintering at elevated temperature. However, almost complete reoxidation of Pd can occur in a fixed‐bed reactor when full CO conversion is reached. These observations highlight the importance of spatially‐resolved operando experiments to draw unambiguous conclusions on the structure–activity relations.[^15^, ^50^, ^51^, ^52^] Complementary surface‐sensitive spectroscopic experiments allowed us to confirm the coexistence of metallic Pd^0^, sub‐oxidized Pd^δ+^ and oxidized Pd^2+^ species in strong interaction with CeO_2_ in the catalyst operating at elevated temperature. Recently, it was shown that coexistence of Pt^0^ and Pt^2+^ in ceria‐supported Pt nanoparticles was due to the presence of Pt^2+^−O−Ce interfacial sites.^[53]^ From the finding that operando DRIFTS spectra did not exhibit any CO adsorbed on highly oxidized Pd sites in the used catalyst, we infer that Pd^2+^−O−Ce moieties detected by X‐ray based spectroscopy are located at the interface between Pd nanoparticles (≈3 nm) and ceria. In line with this, the repeated CO oxidation cycle followed by in situ NAP‐XPS and operando XAS revealed a slightly lower fraction of Pd^2+^−O−Ce fragments due to the increased size of supported nanoparticles and thus lower contribution of interfacial sites (Figures S21–S23).

### Correlating Pd Oxidation States to CO Oxidation Kinetics

**Figure 7:**
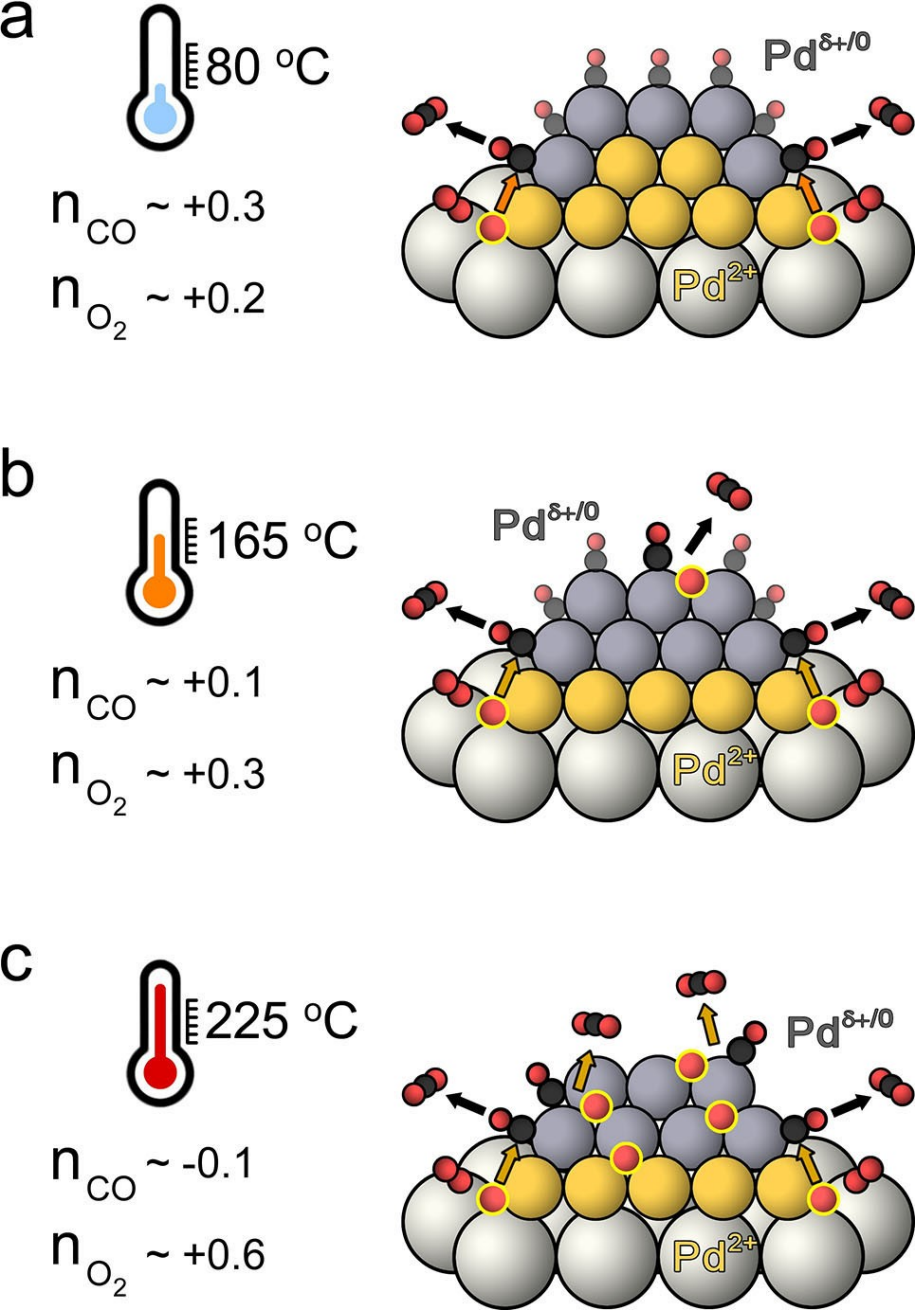
The reaction orders of CO oxidation at a) 80 °C, b) 165 °C, c) 225 °C and schematics of Pd states involved.

A set of in situ and operando spectroscopy experiments demonstrated that Pd is present in several oxidation states in Pd/CeO_2_ catalyst during CO oxidation. Chemical transient experiments showed that metallic Pd species (M−CO) are spectators in low‐temperature CO oxidation, while sub‐oxidized Pd sites (L−CO) in proximity to Pd^2+^−O−Ce interface are most likely the active sites. CO oxidation on these sub‐oxidized Pd species exhibits a different mechanism than the conventional metal‐catalyzed CO oxidation. This is consistent with the positive reaction order in CO (≈0.3), indicating the absence of CO poisoning. Thus, the low‐temperature CO oxidation activity can be linked to the Pd−CeO_2_ interface (Figure 7a). Furthermore, our in situ and operando experiments clearly demonstrated that the Pd speciation changes with reaction conditions. With increasing temperature, a higher fraction of metallic Pd nanoparticles is present, which in turn might influence the apparent reaction kinetics. The reaction orders measured at 80 °C and 165 °C for the Pd/CeO_2_ catalyst (Figure S24) were respectively 0.1 and 0.3 in CO and 0.3 and 0.2 in O_2_. These changes can be attributed to an increased contribution of Langmuir‐Hinshelwood mechanism on metallic Pd[^48^, ^49^] to the global kinetics of CO oxidation at 165 °C (Figure 7b). At even higher temperature of 225 °C, the contribution of metallic sites to the overall activity further increased, as indicated by a negative reaction order in CO (−0.1) and a more positive order in O_2_ (+0.6) (Figure 7c). Hence metallic Pd sites that formed under reaction conditions become important for CO oxidation at elevated temperatures. These results illustrate a complex and dynamic role of different Pd oxidation states during CO oxidation over Pd/CeO_2_ catalysts. These findings also point to limitations of single‐site catalysts for reactions that take place in a broader range of reaction conditions such as encountered in three‐way catalysis. The coexistence of multiple active sites in practical catalysts can benefit the overall performance in a wide operation window.

## Conclusion

In this work we demonstrated that a conventional impregnated Pd/CeO_2_ catalyst contains various Pd‐oxo species, ranging in size from single atoms to clusters and nanoparticles. Using a set of time‐resolved operando and in situ spectroscopic techniques, we assessed the reactivity and stability of these different Pd species under reaction conditions. The oxidized Pd single atoms present in the as‐prepared catalyst display high low‐temperature activity but are prone to reduction under CO oxidation conditions at 80 °C. The in situ formed sub‐oxidized and metallic Pd species display a much lower activity in low‐temperature CO oxidation than oxidized Pd single atoms. The low activity of reduced Pd species can be explained by CO poisoning as demonstrated by operando DRIFTS. The analysis of in situ NAP‐XPS and operando spatially‐resolved XAS data revealed that CO oxidation at elevated temperature causes further reduction and sintering of Pd‐oxo species. This results in the formation of predominantly ≈3 nm Pd nanoparticles with ca. 25 % of Pd atoms in the oxidized state due to strong interaction with CeO_2_. The change in reaction orders as a function of the reaction temperature clearly demonstrates the involvement of metallic Pd in CO oxidation at elevated temperatures. Thus, Pd species that are spectators at low temperature are important to the catalytic performance at higher temperature. This exemplifies the complexity of establishing the structure–activity relationships for technical catalysts that will typically contain different Pd states. The present study shows how a combination of appropriate operando characterization methods can resolve the evolution of active phase under dynamic reaction conditions. Such knowledge is pivotal to the rational design of catalysts.

## Conflict of interest

The authors declare no conflict of interest.

1

## Supporting information

As a service to our authors and readers, this journal provides supporting information supplied by the authors. Such materials are peer reviewed and may be re‐organized for online delivery, but are not copy‐edited or typeset. Technical support issues arising from supporting information (other than missing files) should be addressed to the authors.

Supporting Information

## Data Availability

The data that support the findings of this study are available in the Supporting Information of this article.

## References

[1] F. Meirer , B. M. Weckhuysen , Nat. Rev. Mater. 2018, 3, 324–340.

[2] H. Topsøe , J. Catal. 2003, 216, 155–164.

[3] S. A. Kondrat , J. A. Van Bokhoven , Top. Catal. 2019, 62, 1218–1227.

[4] H. J. Freund , G. Meijer , M. Scheffler , R. Schlögl , M. Wolf , Angew. Chem. Int. Ed. 2011, 50, 10064–10094; 10.1002/anie.201101378 21960461

[5] M. Valden , X. Lai , D. W. Goodman , Science 1998, 281, 1647–1650. 9733505 10.1126/science.281.5383.1647

[6] G. Ertl , Science 1991, 254, 1750–1755. 17829239 10.1126/science.254.5039.1750

[7] A. A. Herzing , C. J. Kiely , A. F. Carley , P. Landon , G. J. Hutchings , Science 2008, 321, 1331–1335. 18772433 10.1126/science.1159639

[8] M. A. Van Spronsen , J. W. M. Frenken , I. M. N. Groot , Chem. Soc. Rev. 2017, 46, 4347–4374. 28589194 10.1039/c7cs00045f

[9] R. J. Farrauto , M. Deeba , S. Alerasool , Nat. Catal. 2019, 2, 603–613.

[10] I. Kocemba , J. Rynkowski , Sens. Actuators B 2011, 155, 659–666.

[11] L. Cao , W. Liu , Q. Luo , R. Yin , B. Wang , J. Weissenrieder , M. Soldemo , H. Yan , Y. Lin , Z. Sun , C. Ma , W. Zhang , S. Chen , H. Wang , Q. Guan , T. Yao , S. Wei , J. Yang , J. Lu , Nature 2019, 565, 631–635. 30700869 10.1038/s41586-018-0869-5

[12] G. S. Zafiris , R. J. Gorte , J. Catal. 1993, 143, 86–91.

[13] V. Pramhaas , M. Roiaz , N. Bosio , M. Corva , C. Rameshan , E. Vesselli , H. Grönbeck , G. Rupprechter , ACS Catal. 2021, 11, 208–214. 33425478 10.1021/acscatal.0c03974 PMC7783867

[14] K. Ding , A. Gulec , A. M. Johnson , N. M. Schweitzer , G. D. Stucky , L. D. Marks , P. C. Stair , Science 2015, 350, 189–192. 26338796 10.1126/science.aac6368

[15] A. M. Gänzler , M. Casapu , D. E. Doronkin , F. Maurer , P. Lott , P. Glatzel , M. Votsmeier , O. Deutschmann , J. D. Grunwaldt , J. Phys. Chem. Lett. 2019, 10, 7698–7705. 31730353 10.1021/acs.jpclett.9b02768

[16] A. M. Gänzler , M. Casapu , F. Maurer , H. Störmer , D. Gerthsen , G. Ferré , P. Vernoux , B. Bornmann , R. Frahm , V. Murzin , M. Nachtegaal , M. Votsmeier , J. D. Grunwaldt , ACS Catal. 2018, 8, 4800–4811.

[17] X. I. Pereira-Hernández , A. DeLaRiva , V. Muravev , D. Kunwar , H. Xiong , B. Sudduth , M. Engelhard , L. Kovarik , E. J. M. Hensen , Y. Wang , A. K. Datye , Nat. Commun. 2019, 10, 1358. 30911011 10.1038/s41467-019-09308-5 PMC6433950

[18] L. DeRita , S. Dai , K. Lopez-Zepeda , N. Pham , G. W. Graham , X. Pan , P. Christopher , J. Am. Chem. Soc. 2017, 139, 14150–14165. 28902501 10.1021/jacs.7b07093

[19] M. Cargnello , V. V. T. Doan-Nguyen , T. R. Gordon , R. E. Diaz , E. A. Stach , R. J. Gorte , P. Fornasiero , C. B. Murray , Science 2013, 341, 771–773. 23868919 10.1126/science.1240148

[20] A. I. Boronin , E. M. Slavinskaya , I. G. Danilova , R. V. Gulyaev , Yu. I. Amosov , P. A. Kuznetsov , I. A. Polukhina , S. V. Koscheev , V. I. Zaikovskii , A. S. Noskov , Catal. Today 2009, 144, 201–211.

[21] R. V. Gulyaev , E. M. Slavinskaya , S. A. Novopashin , D. V. Smovzh , A. V. Zaikovskii , D. Y. Osadchii , O. A. Bulavchenko , S. V. Korenev , A. I. Boronin , Appl. Catal. B 2014, 147, 132–143.

[22] L. Zhang , G. Spezzati , V. Muravev , M. A. Verheijen , B. Zijlstra , I. A. W. Filot , Y.-Q. Su , M.-W. Chang , E. J. M. Hensen , ACS Catal. 2021, 11, 5614–5627. 34055456 10.1021/acscatal.1c00564 PMC8154324

[23] P. Bera , K. C. Patil , V. Jayaram , G. N. Subbanna , M. S. Hegde , J. Catal. 2000, 196, 293–301.

[24] V. Muravev , G. Spezzati , Y.-Q. Su , A. Parastaev , F.-K. Chiang , A. Longo , C. Escudero , N. Kosinov , E. J. M. Hensen , Nat. Catal. 2021, 4, 469–478.

[25] G. Spezzati , Y. Su , J. P. Hofmann , A. D. Benavidez , A. T. DeLaRiva , J. McCabe , A. K. Datye , E. J. M. Hensen , ACS Catal. 2017, 7, 6887–6891. 29034121 10.1021/acscatal.7b02001 PMC5634748

[26] D. Jiang , G. Wan , C. E. García-Vargas , L. Li , X. I. Pereira-Hernández , C. Wang , Y. Wang , ACS Catal. 2020, 10, 11356–11364.

[27] A. K. Datye , H. Guo , Nat. Commun. 2021, 12, 895. 33563970 10.1038/s41467-021-21152-0 PMC7873241

[28] H. Jeong , O. Kwon , B. S. Kim , J. Bae , S. Shin , H. E. Kim , J. Kim , H. Lee , Nat. Catal. 2020, 3, 368–375.

[29] H. Rong , S. Ji , J. Zhang , D. Wang , Y. Li , Nat. Commun. 2020, 11, 5884. 33208740 10.1038/s41467-020-19571-6 PMC7674434

[30] K. Liu , J. Pritchard , L. Lu , R. Van Putten , M. W. G. M. Verhoeven , M. Schmitkamp , X. Huang , L. Lefort , C. J. Kiely , E. J. M. Hensen , E. A. Pidko , Chem. Commun. 2017, 53, 9761–9764. 10.1039/c7cc04759b 28813041

[31] S. Liu , H. Xu , D. Liu , H. Yu , F. Zhang , P. Zhang , R. Zhang , W. Liu , J. Am. Chem. Soc. 2021, 143, 15243–15249. 34495666 10.1021/jacs.1c06381

[32] A. M. Gänzler , B. Betz , S. Baier-Stegmaier , S. Belin , V. Briois , M. Votsmeier , M. Casapu , J. Phys. Chem. C 2020, 124, 20090–20100.

[33] E. D. Goodman , A. C. Johnston-Peck , E. M. Dietze , C. J. Wrasman , A. S. Hoffman , F. Abild-Pedersen , S. R. Bare , P. N. Plessow , M. Cargnello , Nat. Catal. 2019, 2, 748–755. 10.1038/s41929-019-0328-1 PMC7047889 32118197

[34] R. Lang , W. Xi , J.-C. Liu , Y.-T. Cui , T. Li , A. F. Lee , F. Chen , Y. Chen , L. Li , L. Li , J. Lin , S. Miao , X. Liu , A.-Q. Wang , X. Wang , J. Luo , B. Qiao , J. Li , T. Zhang , Nat. Commun. 2019, 10, 234. 30651560 10.1038/s41467-018-08136-3 PMC6335577

[35] Y. Wang , J. Kalscheur , Y.-Q. Su , E. J. M. Hensen , D. G. Vlachos , Nat. Commun. 2021, 12, 5430. 34521852 10.1038/s41467-021-25752-8 PMC8440615

[36] L. Meng , A.-P. Jia , J.-Q. Lu , L.-F. Luo , W.-X. Huang , M.-F. Luo , J. Phys. Chem. C 2011, 115, 19789–19796.

[37] E. M. Slavinskaya , R. V. Gulyaev , A. V. Zadesenets , O. A. Stonkus , V. I. Zaikovskii , Y. V. Shubin , S. V. Korenev , A. I. Boronin , Appl. Catal. B 2015, 166–167, 91–103.

[38] O. A. Stonkus , T. Y. Kardash , E. M. Slavinskaya , V. I. Zaikovskii , A. I. Boronin , ChemCatChem 2019, 11, 3505–3521.

[39] M. Kurnatowska , L. Kepinski , W. Mista , Appl. Catal. B 2012, 117–118, 135–147.

[40] S. Hinokuma , H. Fujii , M. Okamoto , K. Ikeue , M. Machida , Chem. Mater. 2010, 22, 6183–6190.

[41] C. H. Wu , C. Liu , D. Su , H. L. Xin , H. T. Fang , B. Eren , S. Zhang , C. B. Murray , M. B. Salmeron , Nat. Catal. 2019, 2, 78–85.

[42] A. Neitzel , A. Figueroba , Y. Lykhach , T. Skala , M. Vorokhta , N. Tsud , S. Mehl , K. Sevcikova , K. C. Prince , K. M. Neyman , V. Matolin , J. Libuda , J. Phys. Chem. C 2016, 120, 9852–9862.

[43] F. C. Meunier , L. Cardenas , H. Kaper , B. Šmíd , M. Vorokhta , R. Grosjean , D. Aubert , K. Dembélé , T. Lunkenbein , Angew. Chem. Int. Ed. 2021, 60, 3799–3805; 10.1002/anie.202013223 33105066

[44] P. Gelin , A. R. Siedle , J. T. Yates, Jr. , J. Phys. Chem. 1984, 88, 2978.

[45] F. Gao , Y. Wang , D. W. Goodman , J. Am. Chem. Soc. 2009, 131, 5734–5735. 19382813 10.1021/ja9008437

[46] Y. Lu , C. Thompson , D. Kunwar , A. K. Datye , A. M. Karim , ChemCatChem 2020, 12, 1726–1733.

[47] Y. Suchorski , S. M. Kozlov , I. Bespalov , M. Datler , D. Vogel , Z. Budinska , K. M. Neyman , G. Rupprechter , Nat. Mater. 2018, 17, 519–522. 29760509 10.1038/s41563-018-0080-y

[48] A. K. Santra , D. W. Goodman , Electrochim. Acta 2002, 47, 3595–3609.

[49] E. J. Peterson , A. T. DeLaRiva , S. Lin , R. S. Johnson , H. Guo , J. T. Miller , J. Hun Kwak , C. H. F. Peden , B. Kiefer , L. F. Allard , F. H. Ribeiro , A. K. Datye , Nat. Commun. 2014, 5, 4885. 25222116 10.1038/ncomms5885

[50] C. Stewart , E. K. Gibson , K. Morgan , G. Cibin , A. J. Dent , C. Hardacre , E. V. Kondratenko , V. A. Kondratenko , C. McManus , S. Rogers , C. E. Stere , S. Chansai , Y. C. Wang , S. J. Haigh , P. P. Wells , A. Goguet , ACS Catal. 2018, 8, 8255–8262. 30221029 10.1021/acscatal.8b01509 PMC6135604

[51] E. K. Dann , E. K. Gibson , C. R. A. Catlow , V. Celorrio , P. Collier , T. Eralp , M. Amboage , C. Hardacre , C. Stere , A. Kroner , A. Raj , S. Rogers , A. Goguet , P. P. Wells , J. Catal. 2019, 373, 201–208.

[52] J. Singh , M. Nachtegaal , E. M. C. Alayon , J. Stötzel , J. A. Van Bokhoven , ChemCatChem 2010, 2, 653–657.

[53] Y. Li , M. Kottwitz , J. L. Vincent , M. J. Enright , Z. Liu , L. Zhang , J. Huang , S. D. Senanayake , W.-C. D. Yang , P. A. Crozier , R. G. Nuzzo , A. I. Frenkel , Nat. Commun. 2021, 12, 914. 33568629 10.1038/s41467-021-21132-4 PMC7876036

