# Supplementary material for: Operando Spectroscopy Unveils the Catalytic Role of Different Palladium Oxidation States in CO Oxidation on Pd/CeO2 Catalysts
Source: Angew Chem Int Ed Engl. 2022 Apr 5;61(23):e202200434. doi: 10.1002/anie.202200434 (PMC9325467; doi:10.1002/anie.202200434)
Supplement: Supplementary file 1 — Supporting Information [file ANIE-61-0-s001.pdf]

Angewandte Chemie
Eine Zeitschrift der Gesellschaft Deutscher Chemiker

Supporting Information

Operando Spectroscopy Unveils the Catalytic Role of Different Palladium Oxidation States in CO Oxidation on Pd/CeO_2 Catalysts

*V. Muravev, J. F. M. Simons, A. Parastaev, M. A. Verheijen, J. J. C. Struijs, N. Kosinov, E. J. M. Hensen**

Experimental Section

Catalyst preparation

To prepare the Pd/CeO_2 catalyst a commercial ceria powder (Sigma-Aldrich) was loaded with Pd by wet impregnation. An appropriate amount of $Pd(NO_3)_2 \cdot 2H_2O$ (Sigma-Aldrich) was dissolved in 30 mL of ammonia solution (30 wt%, Sigma-Aldrich) at room temperature. The obtained solution was then poured onto ~2 g of ceria powder. The resulting slurry was vigorously stirred till formation of the thick paste. This paste was then dried in air at 110 ºC overnight and subsequently calcined at 300 ºC. Similar procedure was used to obtain Pd/ZrO_2 except for higher calcination temperature (350 ºC). Commercial zirconia powder (Alfa-Aesar) was used as a support.

Catalytic activity measurements

CO oxidation tests were performed in a quartz tube reactor (I.D. 4 mm). Effluent analysis was done by online mass-spectrometry (Balzers TPG-300) and gas chromatography (Interscience Compact GC 4.0), equipped with Plot and Molsieve columns and thermal conductivity detectors (TCDs). Prior to catalytic tests the samples were pressed, crushed, and sieved between 125 µm and 250 µm. Sieved catalyst was then mixed with 250-300 mg of silicon carbide of the same mesh to avoid bypassing of reactants and formation of hot spots. The prepared mixture was then loaded between two quartz wool plugs. All catalysts were pretreated *in situ* under a flow of 20 vol% O_2 in He at 300 ºC for 1 h. The light-off CO oxidation catalytic tests followed by mass-spectrometry were performed from room temperature to 300 ºC at a ramp rate of 5 ºC min^{-1}. The reaction mixture contained 1 vol% CO and 1 vol% O_2 in He at a total flow of 150 mL min^{-1}. Repeated light-off cycles were performed with the same catalyst after cooling to room temperature. CO conversion was estimated based on CO (m/z=28), O_2 (m/z=32) and CO_2 (m/z=44) MS signals and verified by gas chromatography. The reaction rates were plotted in Arrhenius coordinates using the regions of low conversion (<10%). For reaction orders studies an appropriate amount of catalyst was loaded to maintain the CO conversion below 5% at all studied temperatures. Prior to changing the partial pressures of reactants (CO and O_2), the catalyst was stabilized in the reaction feed for at least 14 h to reach the steady state.

Characterization

Powder X-ray diffraction was performed using Bruker Phaser D2 diffractometer equipped with CuKα source. N_2 physisorption at -196 ºC on a TriStar II 3020 apparatus was used to estimate the surface area of the prepared materials using Brunauer-Emmet-Teller method. The Pd loading in ceria and zirconia supported catalysts was determined using ICP-OES instrument (Spectroblue). Raman spectra were acquired using Witec Alpha 300 Raman microscope equipped with 532 nm laser with a power of 2.5 mW and 10X Zeiss lens objective. Transmission electron microscopy was performed using a probe corrected JEOL ARM 200F microscope operated at 200 kV and equipped with a 100 mm^2 Centurio SDD EDX detector. Imaging was performed in HAADF-STEM mode.

Operando XAS experiments were performed at P65 beamline (PETRA III synchrotron, Hamburg). Double crystal Si (311) monochromator and beam size of ~ 0.2 mm x 1.0 mm were used. Spectra were measured at Pd K-edge in fluorescence mode using Si PIPS detector. Simultaneously Pd foil standard was measured in transmission mode to allow reliable energy calibration of the acquired data. Data reduction was performed using Athena software. Pd speciation during operando experiments was determined by linear combination fitting of the XANES data using spectra of Pd foil and powder PdO as standards. EXAFS fitting was

performed in R-space for k^1, k^2, and k^3-weighted data using Artemis software The fitting ranges were Δk = 3-12 Å^{-1} and ΔR = 1.0-3.0 Å. The spectra presented are k^3-weighted and not phase-corrected. Amplitude reduction factors were obtained from fitting of EXAFS spectrum of PdO. For *operando* experiments the catalyst powder was mixed with a sieved fraction (125 µm and 250 µm) of dry BN in a ratio of 1:2. The prepared mixture was fixed between two quartz wool plugs in the quartz reactor (O.D. 3 mm, 0.1 mm wall thickness). The catalyst bed length was about 15 mm. To control sample temperature, the thermocouple was placed inside the reactor in direct contact with the catalyst bed at the outlet side. For temperature control a standard PID thermocontroller (Eurotherm) and two resistive heating elements fixed ~1.5 mm above and below the quartz reactor were used. Unless stated otherwise, the X-ray beam was irradiating the sample near the middle of the catalyst bed. Other parts of the bed were probed by moving the sample stage. For pretreatment, the catalyst was heated from room temperature to 300 ºC with a ramp rate of 5 ºC min^{-1} and dwelled for 30 minutes in 20 vol% O_2 in He. After cooling to 80 ºC a fast switch from 20 vol% O_2 to 1 vol% CO and 1 vol% O_2 in He was performed using a four-way valve equipped with an electric actuator (VICI). The total flow was fixed to 100 mL min^{-1}. The outlet of the reactor was connected to the mass-spectrometer (Pfeiffer Omnistar) and CO (*m/z*=28), O_2 (*m/z*=32) and CO_2 (*m/z*=44) signals were used to calculate the CO conversion. During light-off measurements (80 – 225 ºC) the temperature was ramped at 5 ºC min^{-1}. The same rate for cooling was used. Ex situ XAS measurements at Pd K-edge were performed at the B18 beamline Diamond synchrotron (Didcot). Samples were pressed into pellets and analysed in fluorescence mode using a 9-element Ge detector.

For *in situ* NAP-XPS measurements a SPECS system was used. The monochromated Al Kα X-ray source operated at 20W with a spot size of ~0.3 mm. The differential pumping system of the SPECS Phoibos NAP-150 allows for acquisition of spectra directly in the presence of reactants. To avoid sample charging, catalyst powder was finely grounded and dispersed in ethanol. The obtained suspension was drop casted onto an Al-foil and fixed on a sample holder. After evacuation, the sample was transferred into the NAP-cell and pretreated in 2 mbar of O_2 at 300 ºC. Next the sample was cooled down and reaction mixture of 1 mbar of CO and 1 mbar of O_2 was introduced. Total flow of the gas feed in the cell was 2 mL min^{-1}. Pressure in the cell was regulated by electronic back-pressure controller. All the gasses used during experiments were of high purity (99.999%). The spectra were acquired isothermally upon step-wise heating. Acquisition time for the survey, Pd 3d, Ce 3d, O 1s and C1s spectra for each temperature step was around 2.5 h. A pass energy of 40 eV, step size of 0.1 eV (0.05 eV for Pd 3d) and dwell of 0.5 s were typically used. The binding energy scale was corrected using the U''' component of the Ce 3d core line located at 916.7 eV[1–3]. Shirley background for all spectra. The Pd to Ce surface atomic ratios were estimated using respective atomic sensitivity factors. Photoelectron spectra were fitted using symmetric pseudo-Voigt function, referred to as GL (30) in CasaXPS software. For the Pd 3d spectra containing metallic component, an asymmetric pseudo-Voigt function referred to as LF (0.76, 1.5, 55, 300) in CasaXPS was used. The Ce 3d spectra were fitted according to the models reported earlier[2,4].

Operando DRIFTS experiments were performed using BRUKER Vertex 70v FT infrared spectrometer equipped with a mercury–cadmium–telluride detector and mid-infrared laser source. We used a Praying Mantis accessory and a commercial cell (Harrick). A scheme of the setup is shown in Fig. S23. Catalyst powder (~50 mg, fraction between 125 µm and 250 µm) was placed on top of the quartz wool plug and was in direct contact with sample thermocouple. To allow simultaneous acquisition of catalytic activity data, one of the windows of the dome of the cell was replaced by a steel window with a mounted capillary of the mass-spectrometer (Balzer, Prisma). The capillary of the MS was placed close to the sample surface (3-4 mm above) to ensure efficient gas probing. Gas inlet of the cell was located below the catalyst bed, while the outlet was connected to internal volume of the cell. Using this

configuration, the fast gas replacement (~8-10 s) was achieved during the switches according to MS and IR signals. A home-made gas-delivery system allowed switches between two reaction feeds without significant pressure drops or perturbations of the flow. This was achieved using a four-way valve with two parallel feeds of reactants with equal flow rates (balanced by He and Ar). Electronic back-pressure regulators located at the outlet of the cell and the vent of the four-way valve were set to 200 mbar to prevent the pressure drop upon switches. For *operando* measurements a constant total flow of 100 ml min^{-1} was used. The catalyst was first pretreated in 20 vol% O_2 in He at 300 ºC for 30 minutes. Then the catalyst was cooled down to 80 ºC and the feed was changed to 1 vol% O_2 in He. Several switches between equivalent gas mixtures (e.g., (A) 1%O_2 in He and (B) 1%O_2 in Ar) were performed to obtain a stable catalyst bed that is not perturbed by switches. After stabilization in 1% O_2, a measurement was started and after ~30 s the four-way valve was activated by a remote control to feed the reaction mixture of 1 vol% CO and 1 vol% O_2 in He. Switches to other mixtures were performed in a similar manner. After each switch the catalyst was stabilized under the flow at least for 30 minutes. The rapid-scan mode was used to increase the temporal resolution. To maximize the signal intensity an aperture of 8 cm^{-1} was set. Each spectrum was collected at a resolution of 2 cm^{-1} in the 3780-1000 cm^{-1} range. In each measurement first 300 spectra were acquired by averaging 8 scans with a frequency of 0.6 s^{-1} and the last 100 spectra were collected by averaging 40 scans with a frequency 0.12 s^{-1}. Spectra of the pretreated catalyst were used as a background. All spectra are shown in absorbance. Major IR bands were integrated, plotted over time after normalization and analysed in semi-logarithmic coordinates.

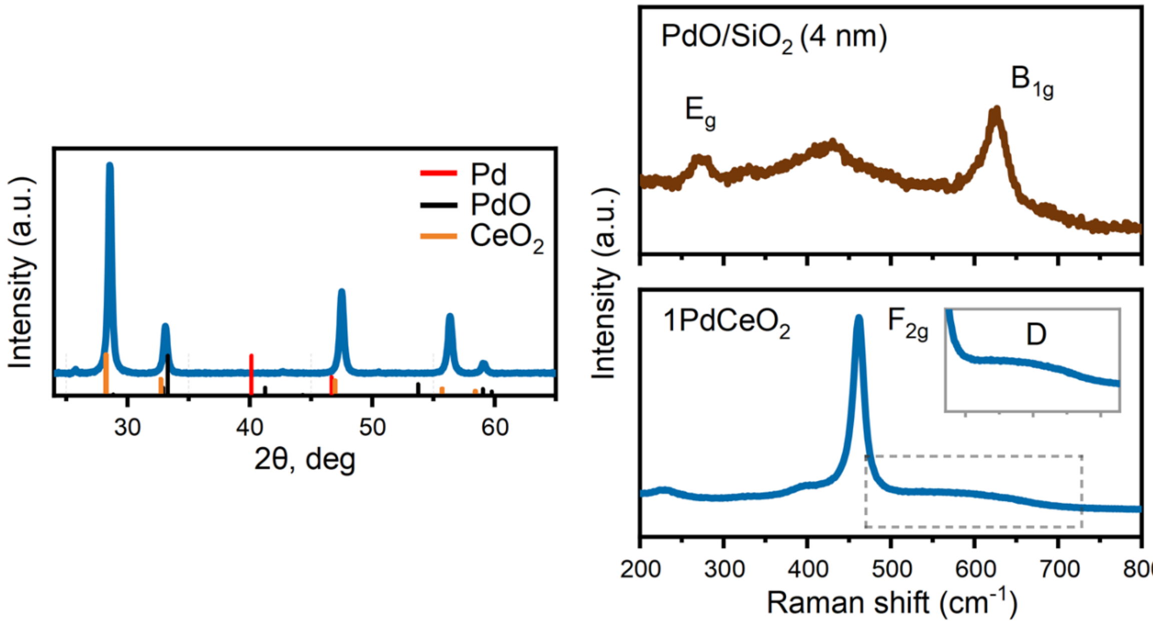

Figure S1. XRD and Raman characterization. No Pd/PdO phases were detected by XRD or Raman spectroscopy. PdO/SiO_2 reference sample was prepared by wet impregnation of a high surface area silica (Sipernat® 50) with palladium nitrate dissolved in deionized water, dried, and calcined at 300 ºC. The size of PdO domains on silica are ~4 nm in size as estimated from XRD data by Scherrer equation. The vibrational bands of PdO (E_g and B_{1g}) were not found in Raman spectrum of Pd/CeO_2 which suggests that Pd species are highly dispersed.

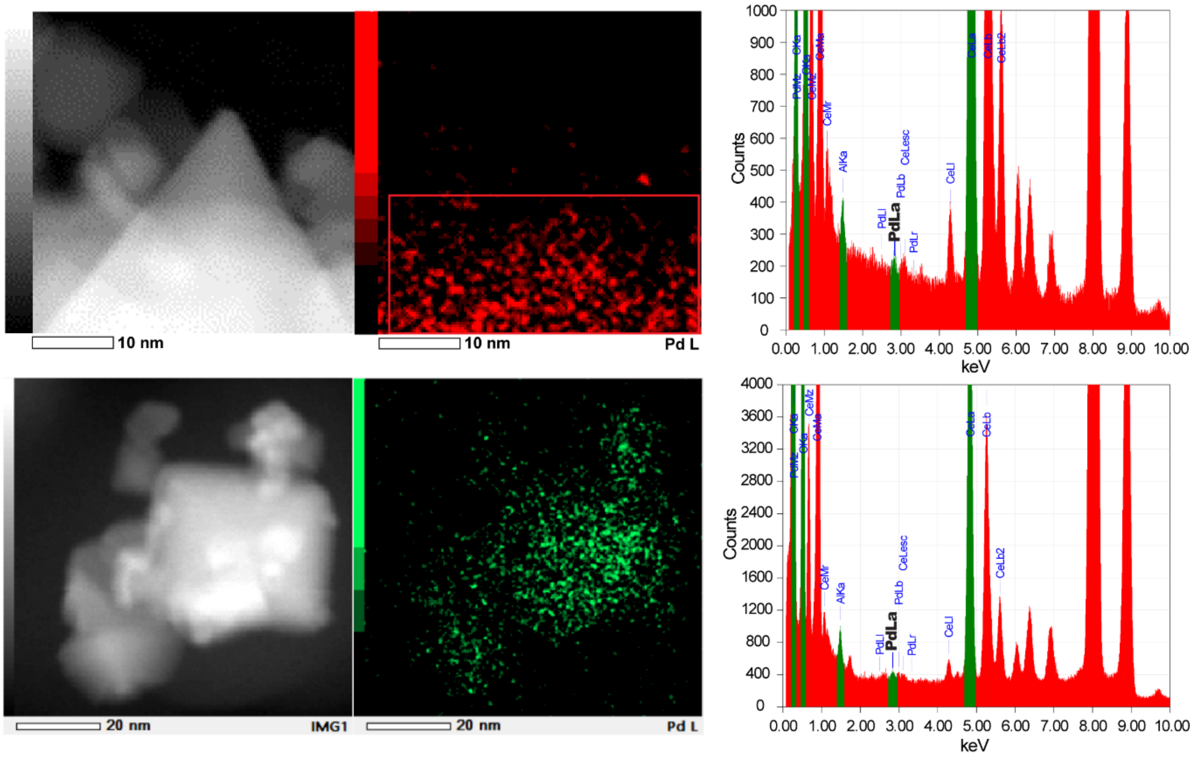

Figure S2. HAADF-STEM images and EDX spectroscopy data obtained for fresh Pd/CeO_2 sample. EDX data indicates that part of Pd is present in the form of highly dispersed species that cannot be identified by HAADF-STEM. In the regions where no particles/clusters of Pd can be seen, EDX spectra displays Pd-related components. Together with the images shown above these results suggest that in the as-prepared catalyst Pd nanoparticles, clusters and highly-dispersed species coexist with each other.

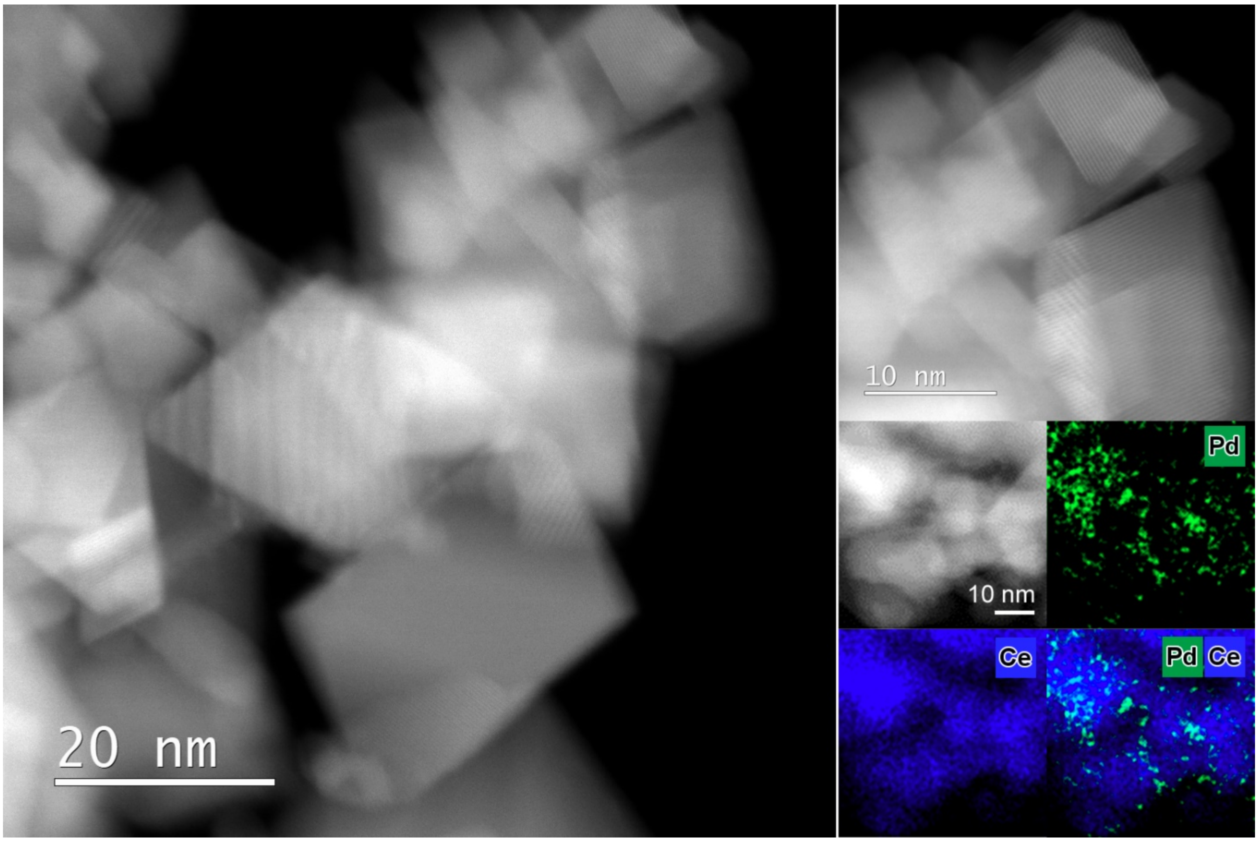

Figure S3. HAADF-STEM and EDX mapping images of fresh Pd/CeO_2 sample. Regions of homogeneous distribution of Pd over CeO_2 can be seen. Some clustered Pd species can also be identified.

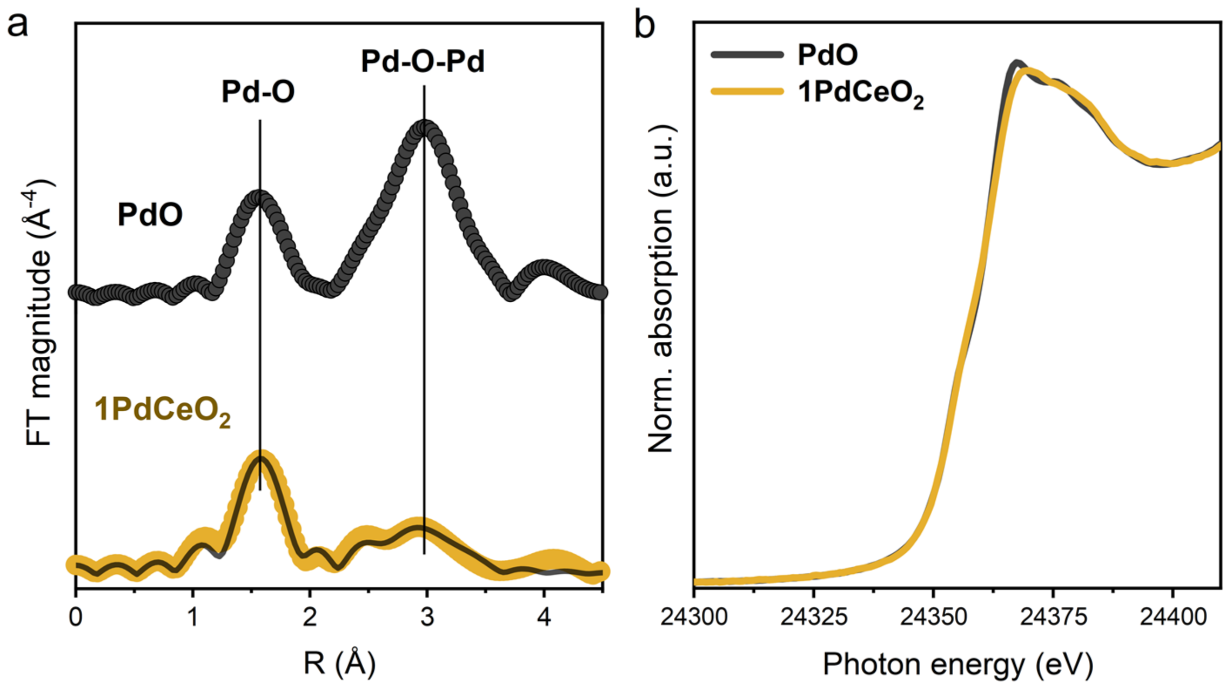

Figure S4. Ex situ XAS of fresh Pd/CeO_2 sample and PdO reference (Sigma-Aldrich): FT-EXAFS in R-space (a), XANES region (b). In contrast to PdO, the second shell scattering is diminished in Pd/CeO_2 sample, which indicates a high dispersion of Pd-oxo species. Details of the fit shown in Table S1. XANES region manifests a slight shift of the white-line maximum to higher energies for spectra of Pd/CeO_2 sample. The shape of the XANES spectrum of Pd/CeO_2 sample also differs from PdO, implying that electronic state of Pd is different, likely due to the strong interaction with ceria. The average oxidation state is close to Pd^{2+}.

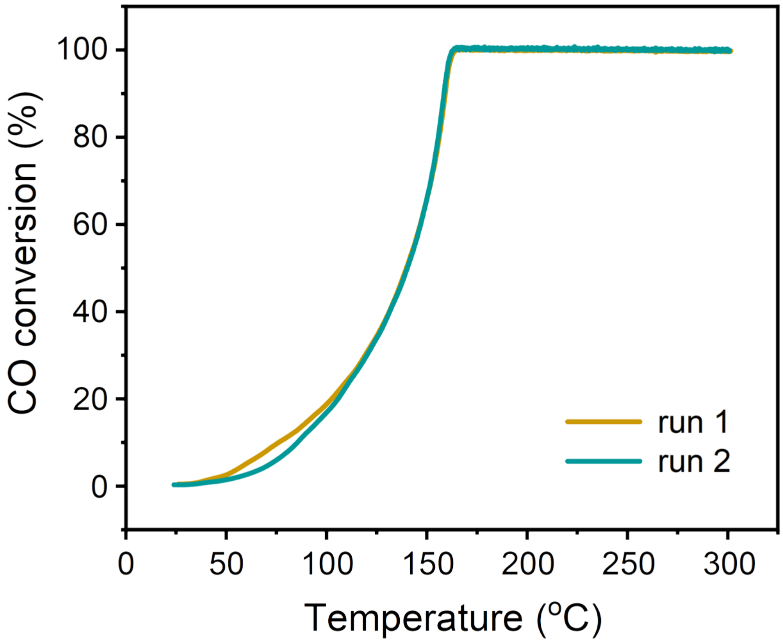

Figure S5. CO oxidation light-off measurements for Pd/CeO_2 sample. Sieved fraction (125 to 250 µm) was diluted with ~250 mg of SiC. The catalyst was pretreated in 20% O_2/He at 300 ºC for 1h. Reaction feed: 1% CO + 1% O_2 in He, ramp rate 5 ºC/min. Flow rate: 150 ml/min and total GHSV of 180,000 ml/g_{cat} h^{-1}.

Note S1. Standards for linear combination fitting (LCF) analysis of operando XANES data.

As shown in Figure S6a,c, the XANES features as well as EXAFS of bulk PdO are significantly different from those of oxidized Pd species in Pd/CeO_2 catalyst. Therefore, the XANES spectrum of the pretreated catalyst (20% O_2, 300 °C) exposed to 1%O_2 at 80 °C was used as a standard for linear combination fitting analysis of the XANES data representing the initial oxidized state of Pd^{2+}. Figure S6b,d shows that also the XANES and EXAFS of bulk metallic Pd (foil) significantly differ from those of reduced Pd species in Pd/CeO_2 catalyst obtained by exposure to two consecutive light-off runs (80 °C to 225 °C) and measured at the inlet/start of the catalyst bed. According to EXAFS fitting (Table S1, Fig. S33), no Pd-O shell was found, confirming the reduced state of Pd. The Pd-Pd coordination numbers for reduced Pd/CeO_2 are lower than for bulk Pd metal (~10 vs. 12), suggesting the presence of metallic Pd in the form of nanoparticles. Since XANES features of spectra for metal nanoparticles are known to be different from their bulk counterparts, we used XANES spectrum of such a reduced Pd/CeO_2 catalyst for LCF analysis to more accurately represent the Pd^0 state. The use of spectra of dispersed Pd^{2+} and Pd^0 species as standards for LCF analysis results in a good fit of individual XANES spectra obtained during operando measurements (Fig. S26-S29).

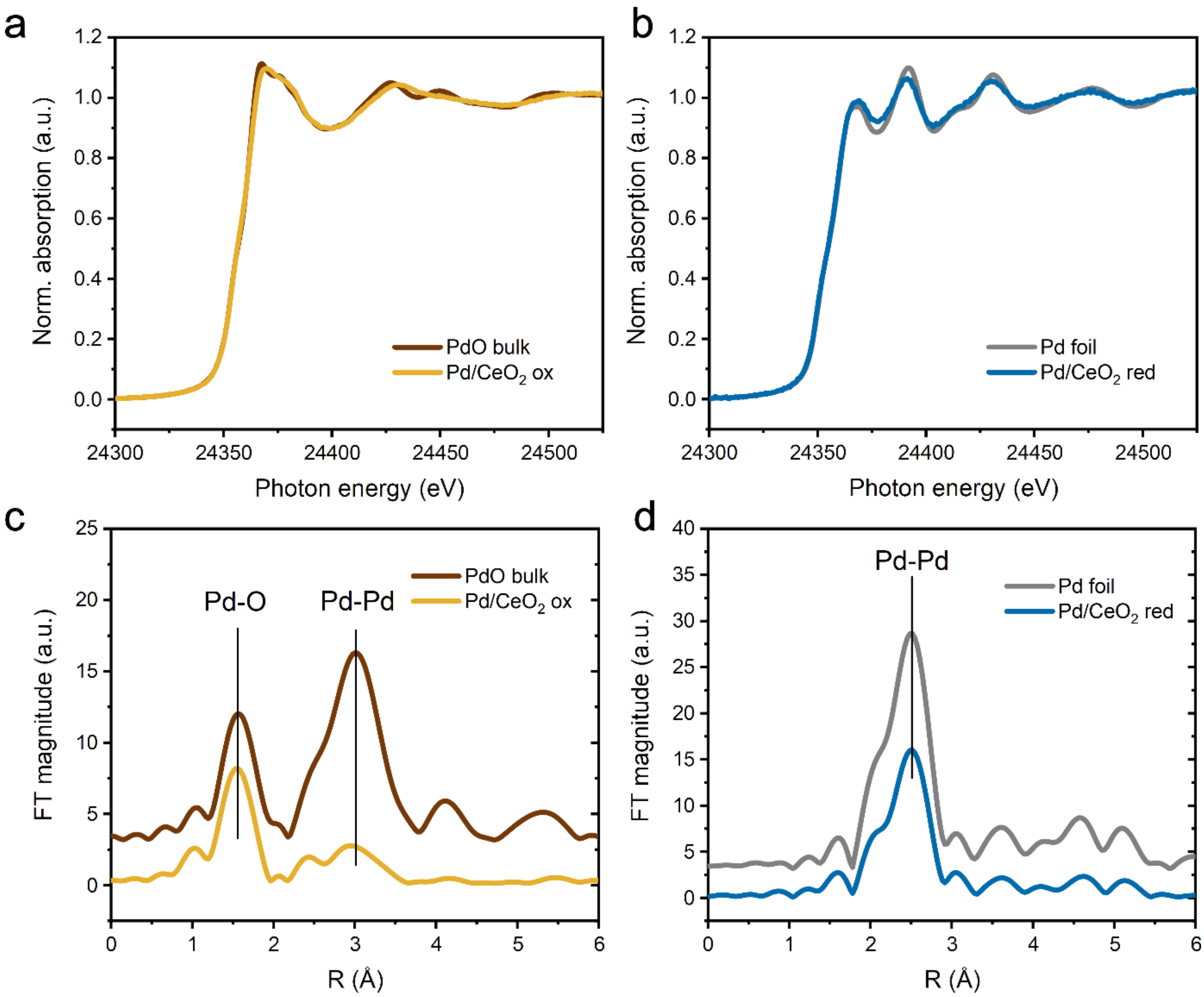

Figure S6. XANES and EXAFS data for (a, c) bulk PdO and pretreated – "oxidized" Pd/CeO_2 catalyst with highly dispersed Pd; (b,d) bulk Pd and used – "reduced" Pd/CeO_2 catalysts with metallic Pd nanoparticles. The XANES spectra of oxidized and reduced states of Pd/CeO_2 were used as standards for LCF instead of spectra of bulk Pd and PdO.

Note S2. Fluctuation of CO conversion during operando XAS experiments.

The observed fluctuations of CO conversion during the first minutes after the switch from O_2 to $CO+O_2$ feed were caused by the superposition of several factors. The reaction of CO oxidation is highly exothermic, and the produced heat can effectively sustain the catalytic reaction and cause temperature fluctuations inside the catalyst bed[5]. In the first instances after the switch the catalyst is exposed to reaction conditions and is highly reactive, due to the majority of Pd still being oxidized and well-dispersed. The produced heat is then sufficient to maintain the reaction till the point at which a certain fraction of Pd sites becomes reduced and CO conversion drops. During this stage the heating rods of the reactor setup (resistively heated wires wrapped around the ceramic cylinders) rapidly cool down, since the thermocouple, inserted in the middle of the bed, reads an increase in temperature caused by the exothermicity of the reaction. Once the conversion drops, due to the reduction of Pd, readings of the thermocouple decrease and then the heating unit starts to heat up the catalyst bed. Since the processes of ignition-extinction of the reaction are quite fast, the latency in the response of the heating system causes the mismatches between cooling-heating cycles of the catalyst bed and of the heating rods, which in turn lead to the observed fluctuation. As will be shown later these effects are also enhanced due to the reoxidation of Pd (and possible recovery of the active sites), when high CO conversion levels are reached. We should note, that high space velocities (100 ml/min, GHSV ~ 200,000 ml/g_{cat} h^{-1}) and dilution of the catalyst with a sieved fraction of BN (catalyst : BN = 1:2 by weight) were used to avoid the formation of hot spots and mimic the kinetic measurements. We assume that the main difference of the reactor cell used for XAS measurements which caused the observed phenomena is related to the heating system that is not thermally isolated from ambient air and is controlled by a thermocouple placed inside the bed. In our conventional reactor the thermocouple is located next to the quartz reactor. The slower response of this system to the overheating of the catalyst bed results in the absence of such fluctuations.

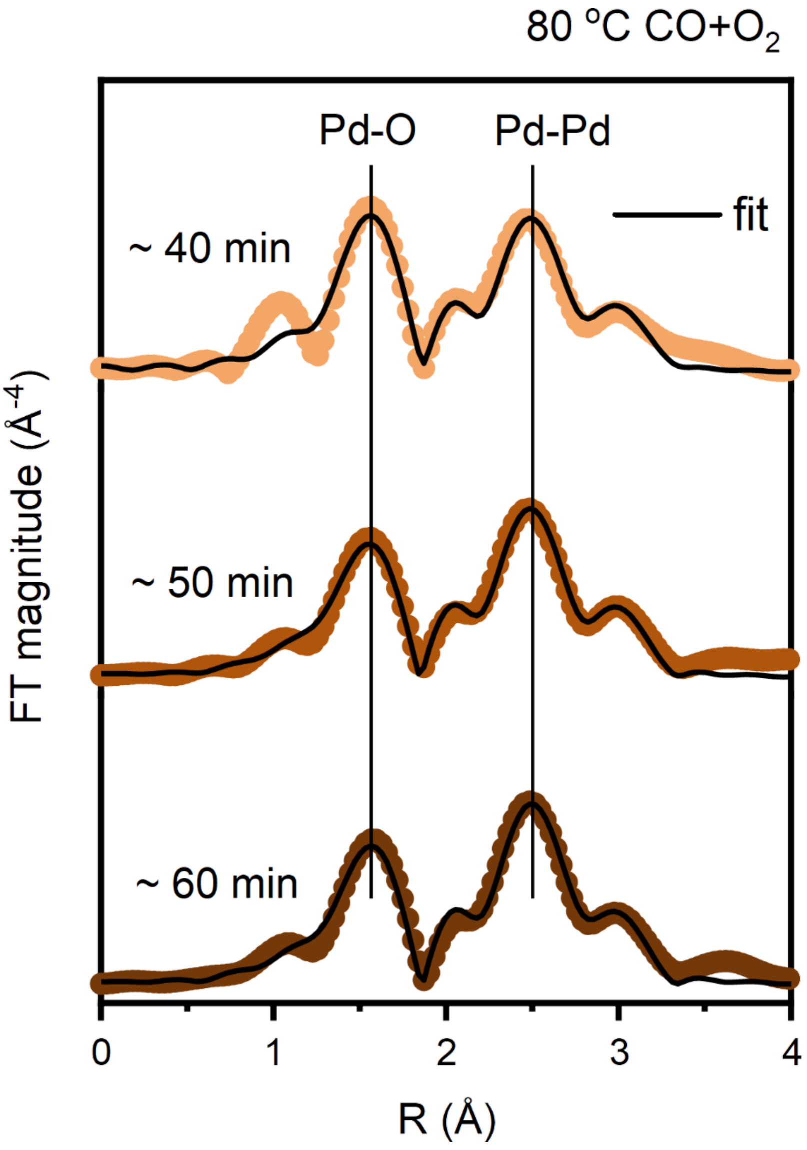

Figure S7. R-space EXAFS spectra of the Pd/CeO_2 sample acquired in the middle of the catalyst bed during stabilization in the reaction mixture after the switch from O_2 to CO+O_2 at 80 °C. Fitting results shown in Table S1. The contribution of Pd-Pd scattering related to metallic Pd increases as a function of time after the switch suggesting sintering of Pd (see Table S1 for details of the fit), whereas LCF analysis shows relatively small changes in Pd^{2+}/Pd^0 ratio. Accordingly, we propose that the loss of the activity in low-temperature CO oxidation follows two steps: a fast one (first minutes of reaction) primarily due to reduction of highly dispersed Pd-O species (Pd^{2+} to Pd^0) and a slower one, due to agglomeration of Pd into larger nanoparticles.

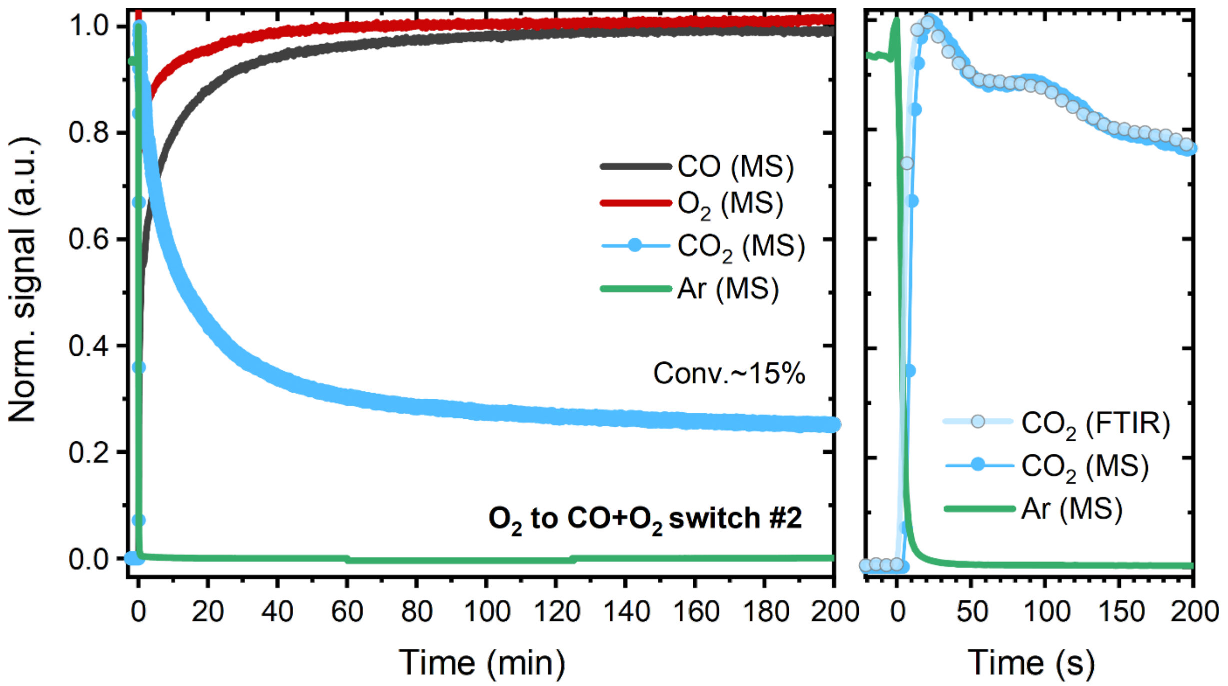

Figure S8. Mass-spectrometry data (left panel) acquired during chemical transient from O_2 to $CO+O_2$ mixture at 80 ºC followed by operando DRIFTS on Pd/CeO_2 catalyst. As shown on the right panel the integrated IR signal of CO_2 band overlaps with the CO_2-related signal from MS data (*m/z* = 44). Gas holdup time ~ 8 s (*m/z* = 40, Ar). Conditions: "O_2" – 1% O_2 in He; "$CO+O_2$" – 1% CO + 1% O_2 in He, total flow 100 ml/min.

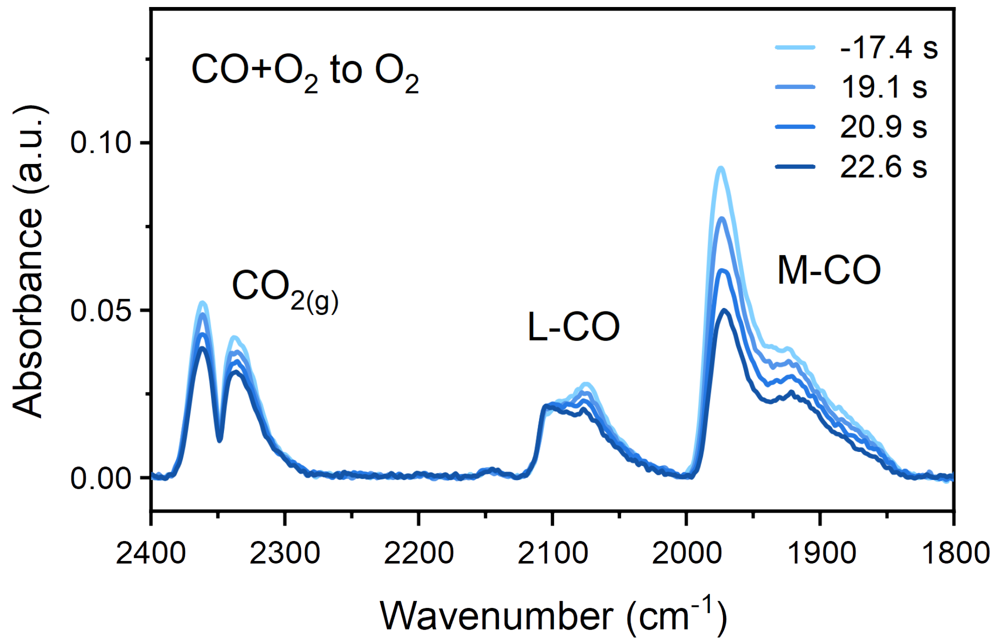

Figure S9. Operando DRIFTS spectra of Pd/CeO_2 sample acquired during chemical transient from CO+O_2 to O_2 mixture at 80 ºC. In this time period, the removal of linear carbonyls was slowed down, while the rate of removal of bridged and hollow carbonyls increased substantially. As explained in the main text, possible interconversion between carbonyls and ignition of the reaction at metallic sites due to facile oxygen dissociation are the likely reason for this phenomena.

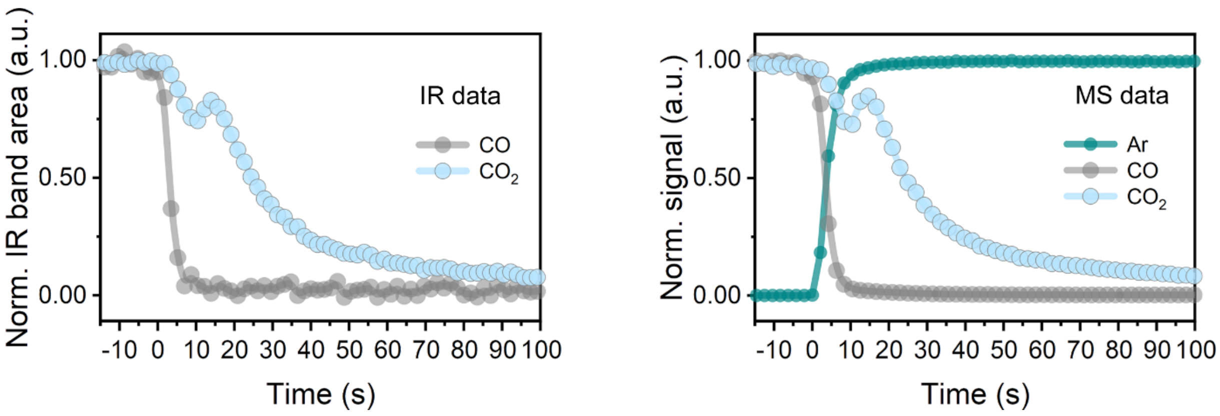

Figure S10. The extra peak of CO_2 formation, related to rapid oxidation of CO on metallic sites at low CO coverage, is seen both in IR and MS data upon switch from $CO+O_2$ to O_2. The MS and IR data on gas composition are very similar, confirming the rapid gas-phase replacement under applied conditions.

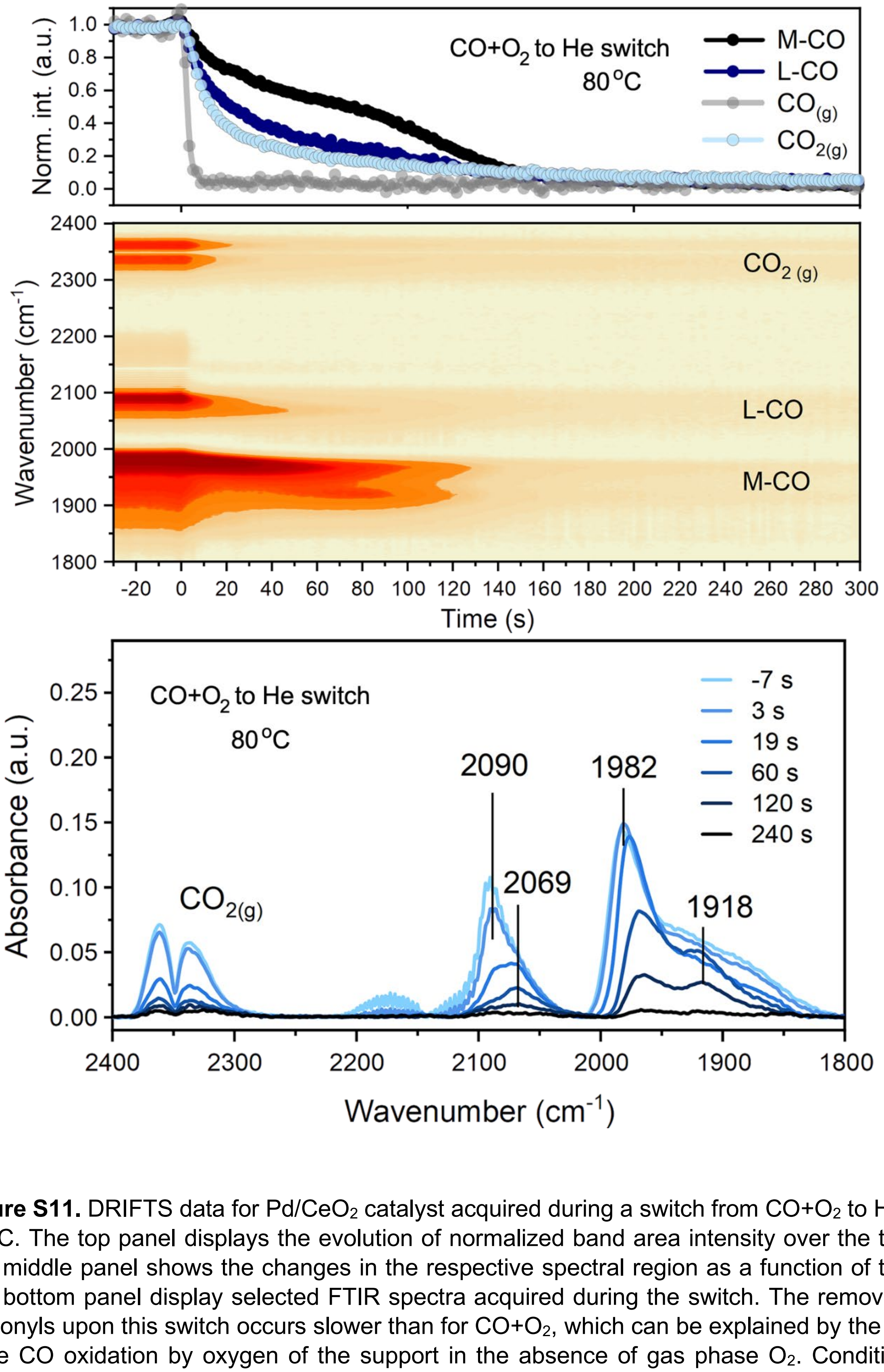

Figure S11. DRIFTS data for Pd/CeO_2 catalyst acquired during a switch from CO+O_2 to He at 80 ºC. The top panel displays the evolution of normalized band area intensity over the time. The middle panel shows the changes in the respective spectral region as a function of time. The bottom panel display selected FTIR spectra acquired during the switch. The removal of carbonyls upon this switch occurs slower than for CO+O_2, which can be explained by the less facile CO oxidation by oxygen of the support in the absence of gas phase O_2. Conditions: "CO+O_2" – 1% CO + 1% O_2 in He; total flow 100 ml/min.

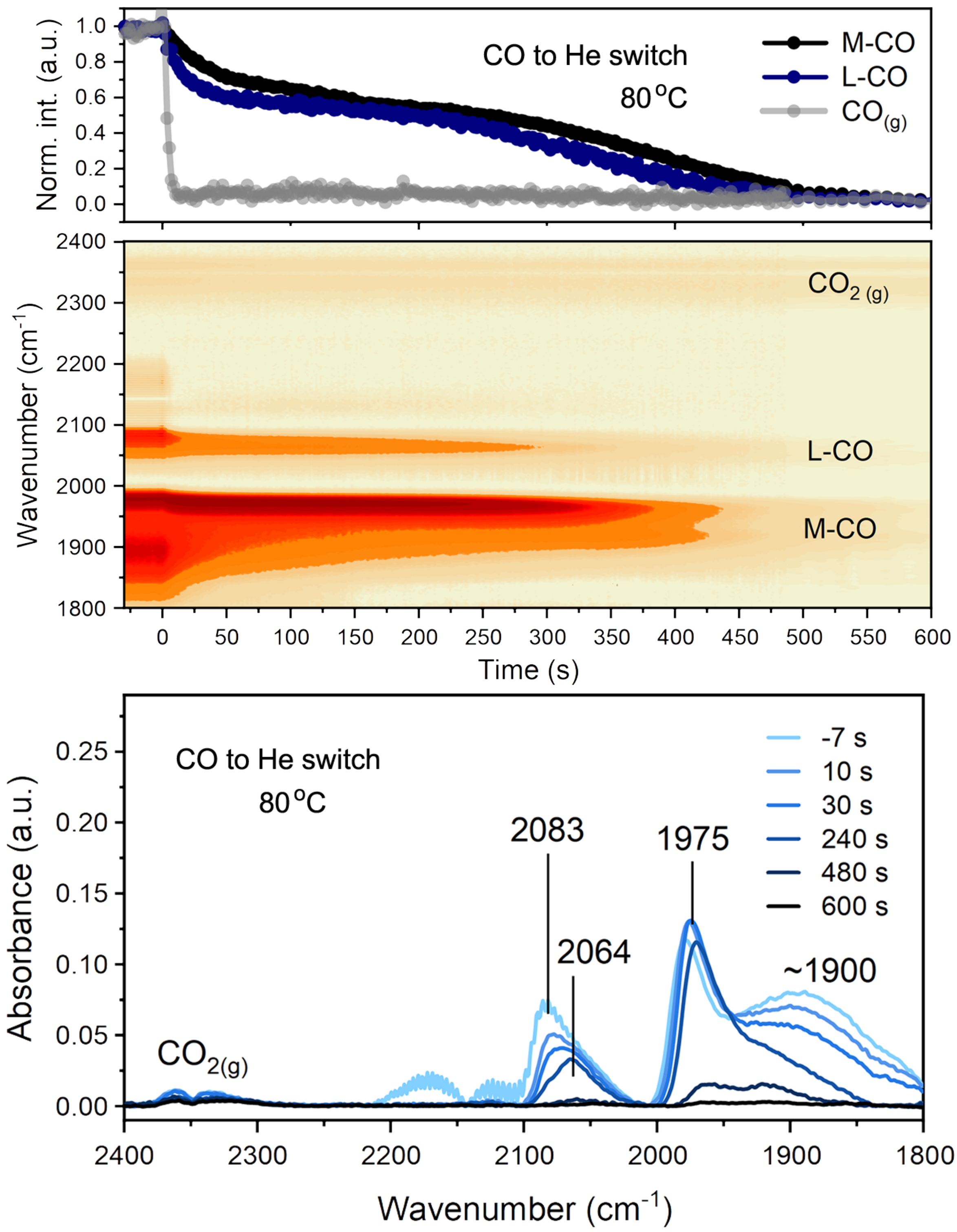

Figure S12. DRIFTS data for Pd/CeO_2 catalyst acquired during a switch from CO to He at 80 ºC. The top panel displays the evolution of normalized band area over the time. The middle panel shows the changes in the respective spectral region as a function of time. The bottom panel display selected FTIR spectra acquired during the switch. During exposure to pure CO the oxygen was depleted from the catalyst, as seen by the CO_2 band. Once the switch to He was performed, CO_2 signal rapidly vanished. The carbonyl removal over oxygen-depleted catalyst occurs much longer than in case CO+O_2 to He switch. This points to a key role of oxygen provided by the ceria support in low-temperature CO oxidation. Conditions: "CO" – 1% CO in He; total flow 100 ml/min.

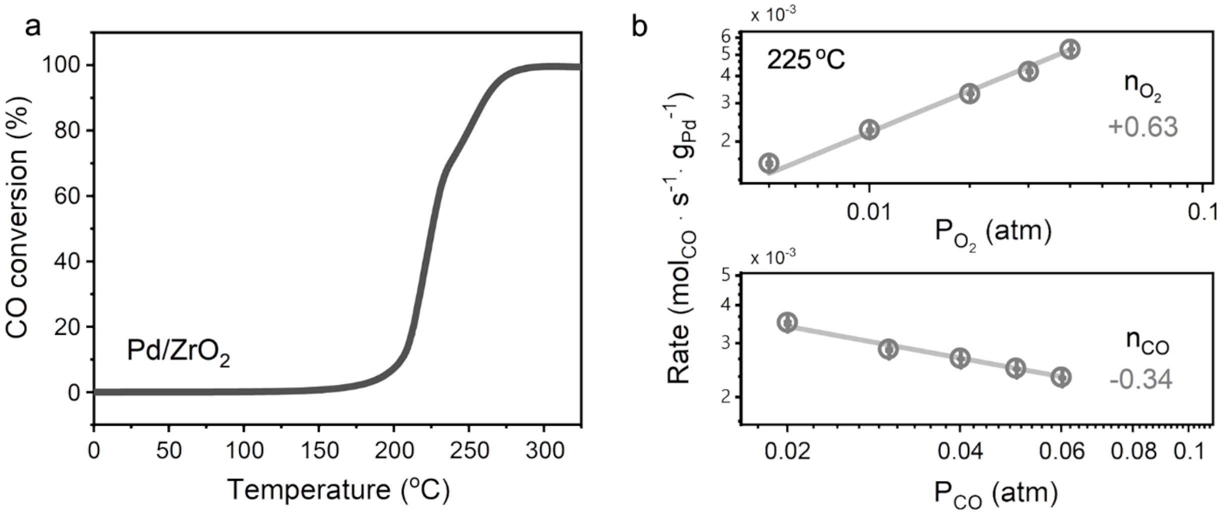

Figure S13. CO oxidation light-off measurements for Pd/ZrO_2 sample (a). Sieved fraction (125 to 250 μm) was diluted with ~250 mg of SiC. The catalyst was pretreated in 20% O_2/He at 300 ºC for 1h. Reaction feed: 1% CO + 1% O_2 in He, ramp rate 5 ºC/min. Flow rate: 150 ml/min and total GHSV of 180,000 ml/g_{cat} h^{-1}. (b) Reaction orders measurements. The catalyst was stabilized on the stream for ~ 16h at 225 ºC to reach steady state. The lower partial pressures of CO were not used for estimation of reaction orders due to high CO conversion levels (>15%) in such lean conditions. Pd/ZrO_2 catalyst displays much lower low-temperature CO oxidation activity than Pd/CeO_2 catalyst. As stems from reaction orders study, Pd/ZrO_2 follows Langmuir-Hinshelwood CO oxidation kinetics typical for metallic active sites.

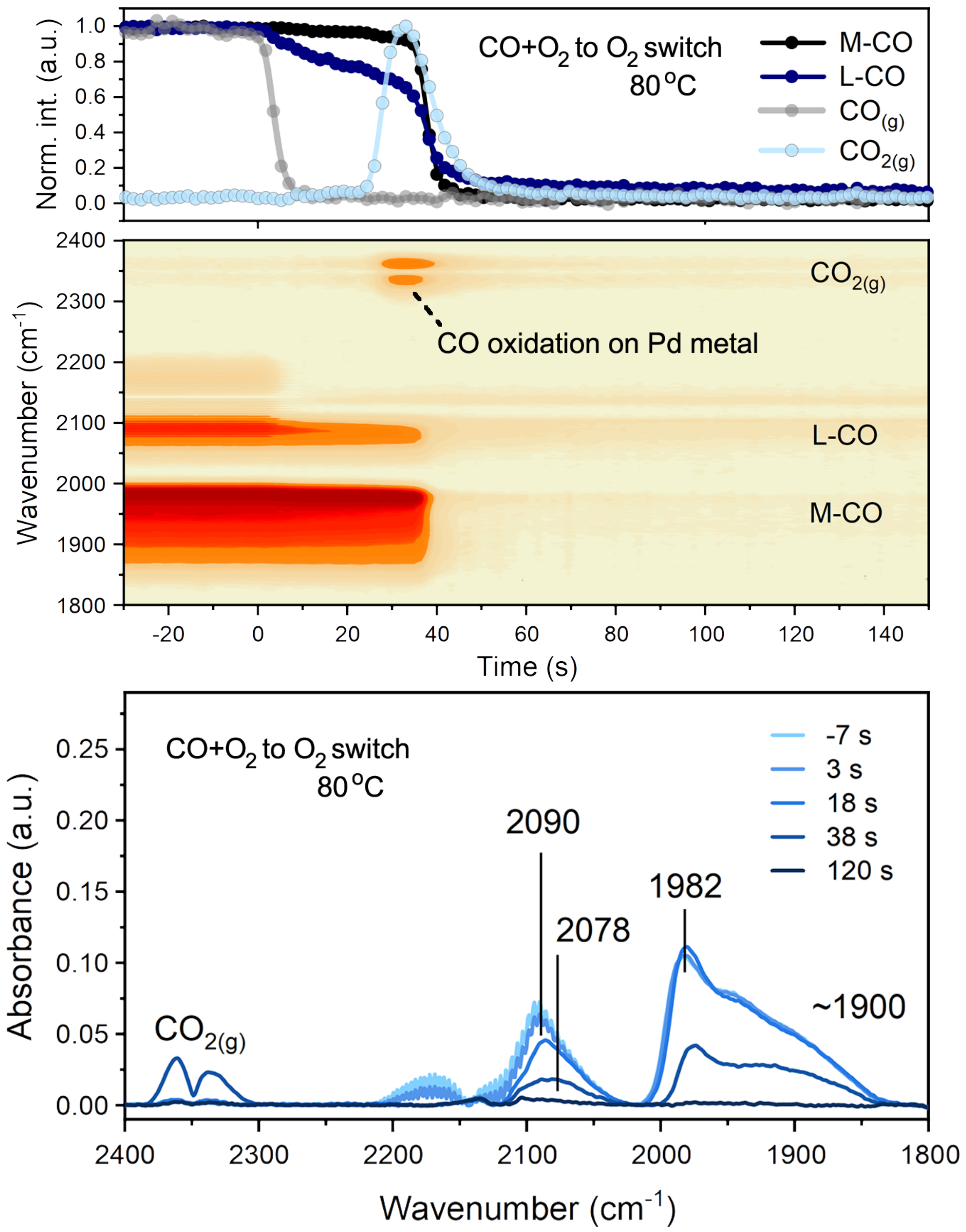

Figure S14. DRIFTS data for Pd/ZrO_2 catalyst acquired during a switch from CO+O_2 to O_2 at 80 ºC. The top panel displays the evolution of normalized band area intensity over the time. The middle panel shows the changes in the respective spectral region as a function of time. The bottom panel display selected FTIR spectra acquired during the switch. In contrast to Pd/CeO_2, the Pd/ZrO_2 catalyst is nearly inactive at 80 ºC. The positions of CO bands are, however, similar to the ones observed for ceria-supported catalyst. In contrast to Pd/CeO_2, CO oxidation over Pd/ZrO_2 takes place only in pure O_2 (at ~35s) due to poisoning of metallic sites by CO and absence of interface capable of providing reactive oxygen. Conditions: “CO+O_2” – 1% CO and 1% O_2 in He; “O_2” – 1% O_2 in He, total flow 100 ml/min.

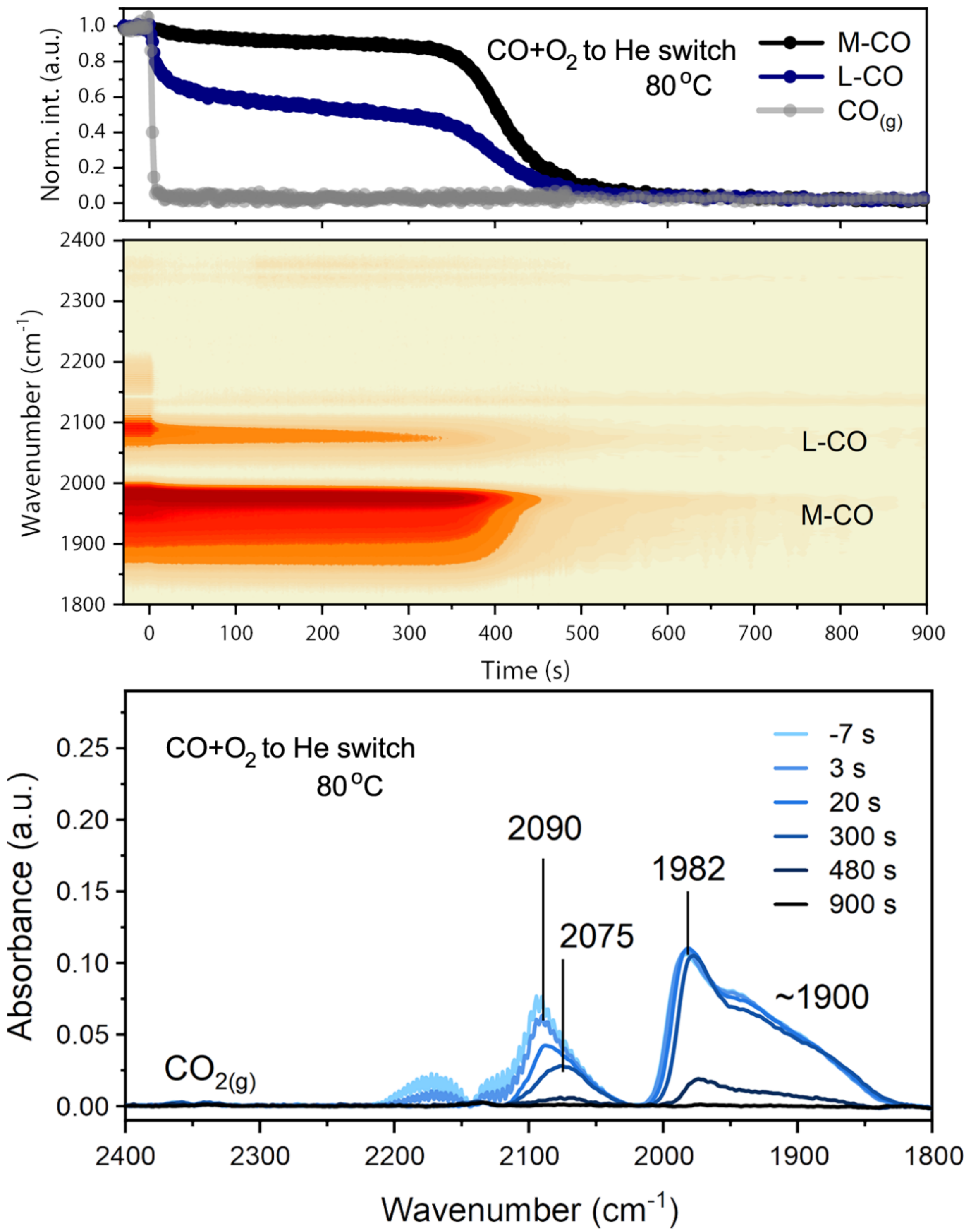

Figure S15. DRIFTS data for Pd/ZrO_2 catalyst acquired during a switch from $CO+O_2$ to He at 80 ºC. The top panel displays the evolution of normalized band area intensity over the time. The middle panel shows the changes in the respective spectral region as a function of time. The bottom panel display selected FTIR spectra acquired during the switch. In contrast to Pd/CeO_2, the removal of carbonyls from Pd/ZrO_2 catalyst is much slower. Conditions: "$CO+O_2$" – 1% CO and 1% O_2 in He, total flow 100 ml/min.

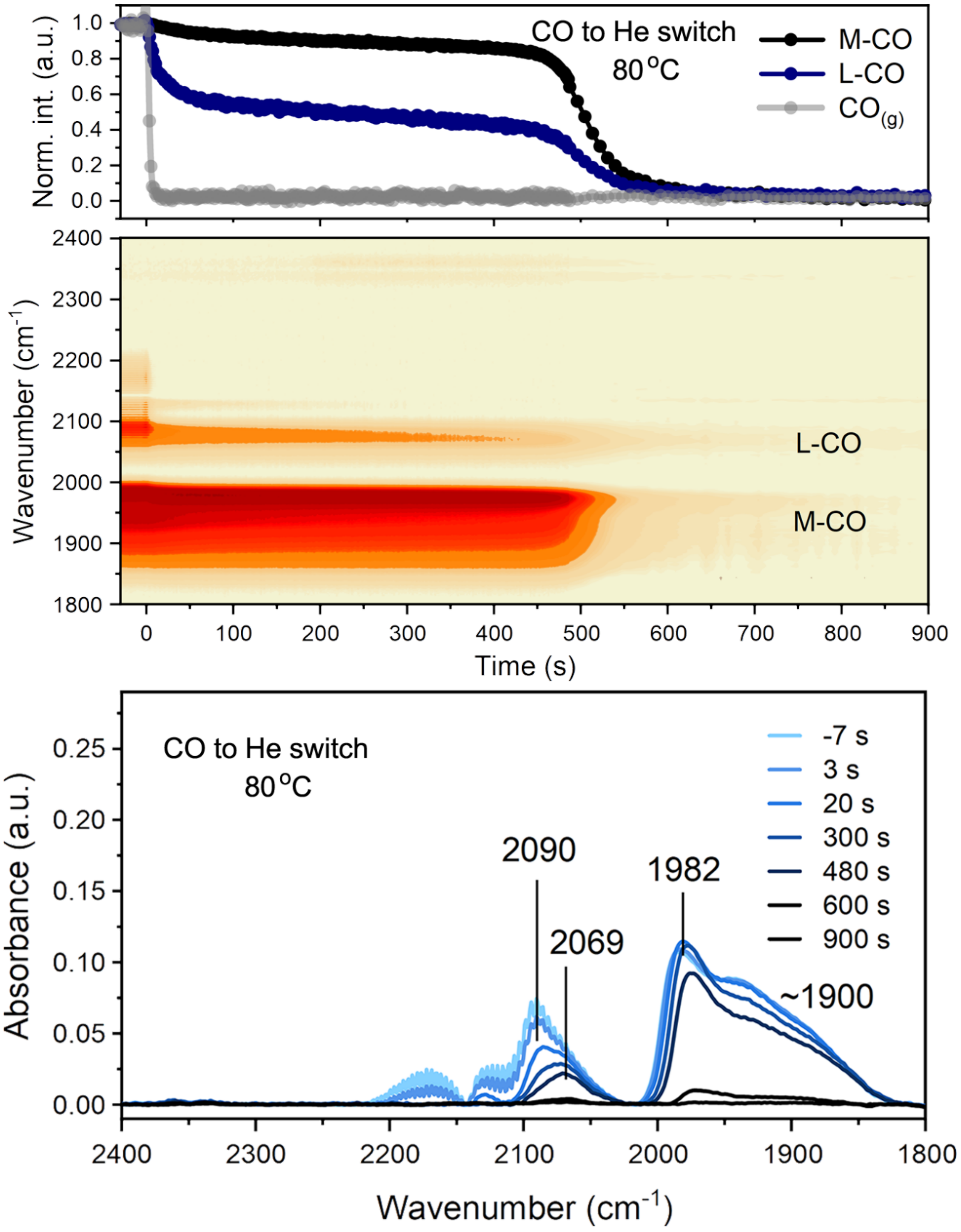

Figure S16. DRIFTS data for Pd/ZrO_2 catalyst acquired during a switch from CO to He at 80 ºC. The top panel displays the evolution of normalized band area intensity over the time. The middle panel shows the changes in the respective spectral region as a function of time. The bottom panel display selected FTIR spectra acquired during the switch. In stark contrast to Pd/CeO_2, only a negligible signal of CO_2 was observed during exposure of Pd/ZrO_2 to pure CO. This implies that the amount of reactive oxygen in Pd/ZrO_2 sample is low. Furthermore, the kinetics of carbonyl disappearance is similar during CO+O_2 to He and CO to He switches, suggesting that in the absence of active interfacial sites pre-exposure to pure CO have little influence on the reactivity of adsorbed CO. This finding further supports the idea of ceria oxygen participation in CO oxidation on Pd/CeO_2 catalysts. Conditions: “CO+O_2” – 1% CO and 1% O_2 in He, total flow 100 ml/min.

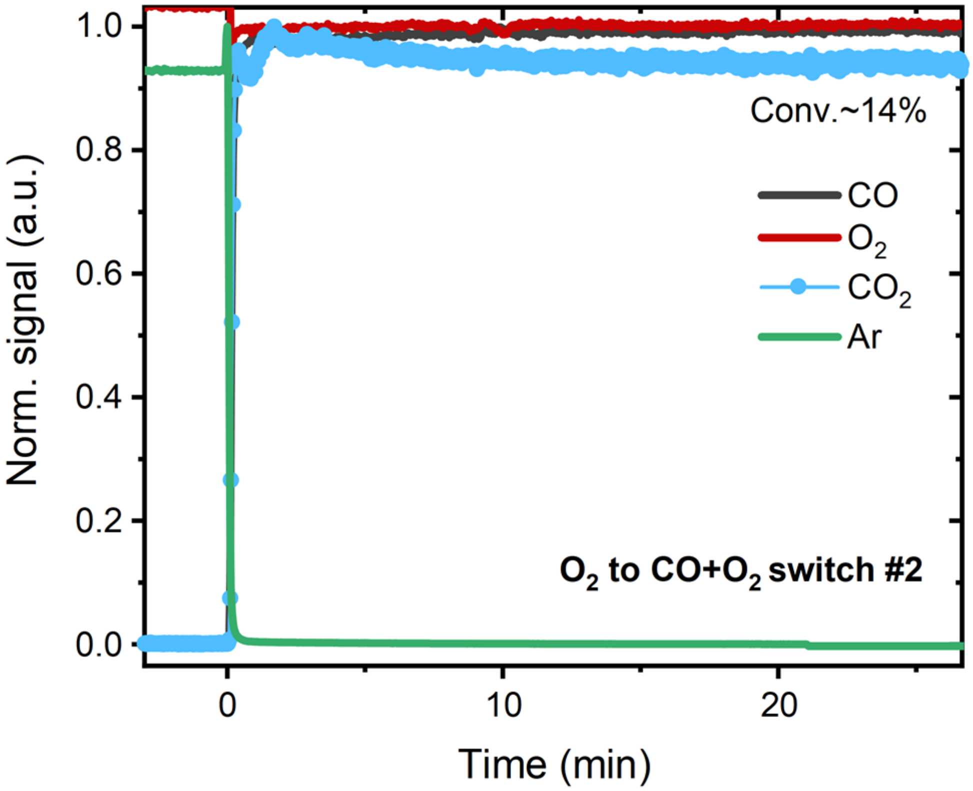

Figure S17. Mass-spectrometry data acquired during second chemical transient from O_2 to $CO+O_2$ mixture at 80 ºC over Pd/CeO_2 catalyst followed by operando DRIFTS. Gas holdup time ~ 8 s (Ar). Conditions: "O_2" – 1% O_2 in He; "$CO+O_2$" – 1% CO + 1% O_2 in He, total flow 100 ml/min. The absence of transient high reactivity towards CO oxidation, which was seen during the first switch, is due to the absence of highly dispersed and/or single-atom Pd-oxo species. Apparently, these species cannot be regenerated in pure O_2 at 80 ºC.

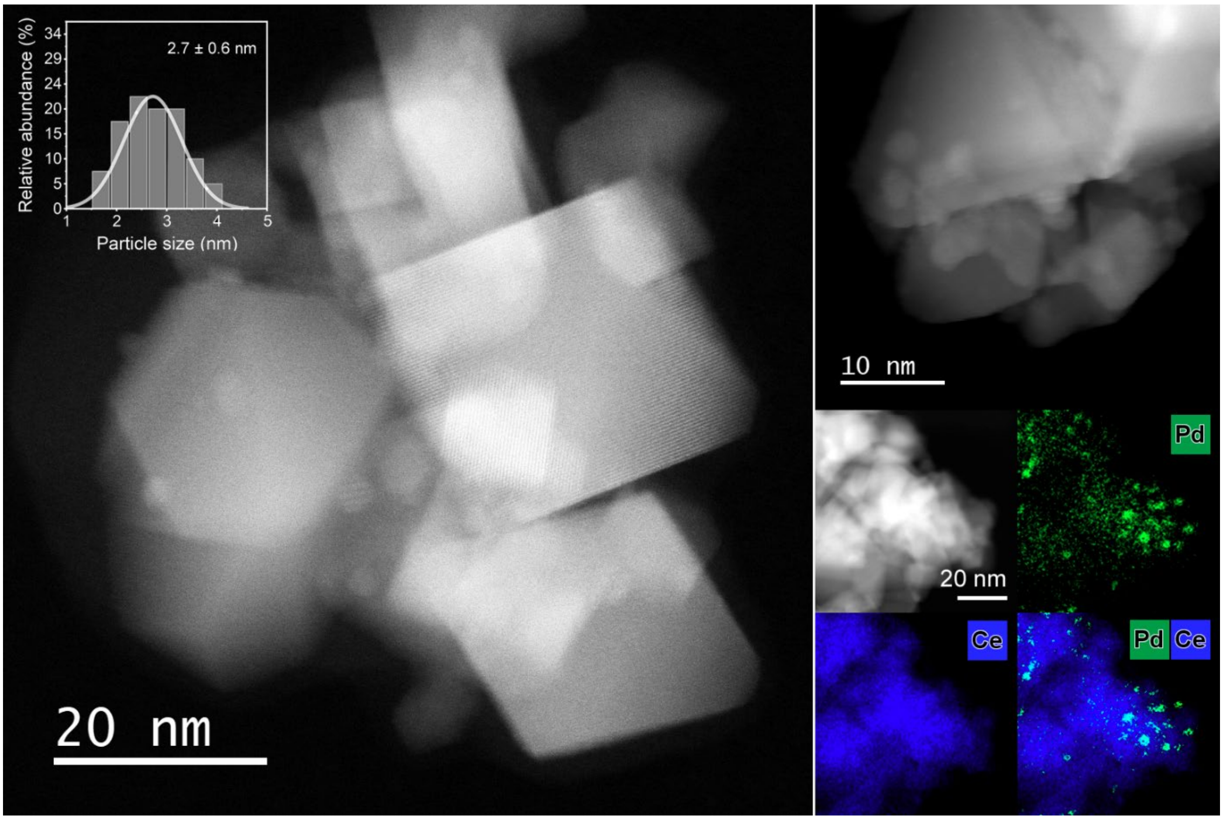

Figure S18. HAADF-STEM and EDX mapping images of used Pd/CeO_2 sample. Most of Pd is found in the form of small Pd-PdO nanoparticles of ~ 3 nm size.

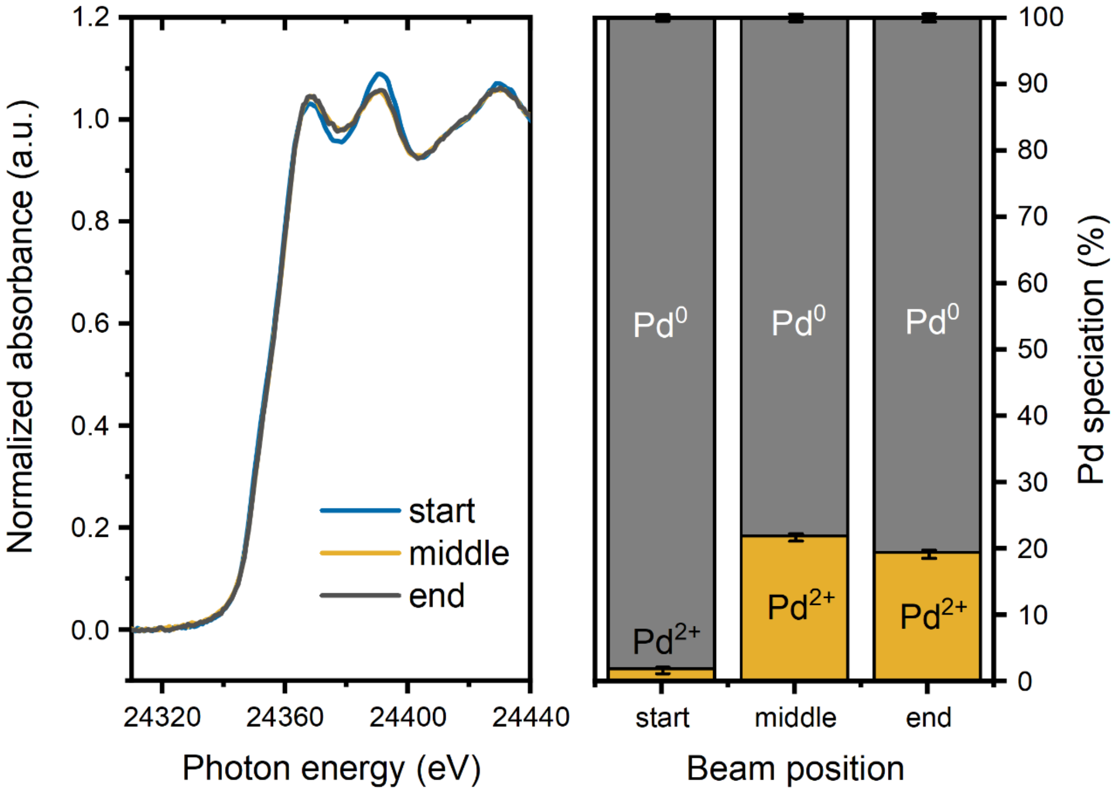

Figure S19. Operando XANES spectra at Pd K-edge acquired for Pd/CeO_2 sample during CO oxidation at 80 ºC after the first light-off cycle (80 to 225 ºC). Spectra were measured in different parts of the catalyst bed. As can be seen the inlet of the catalyst bed is more reduced than the rest of the catalyst likely due to the CO concentration gradients present during reaction at high conversion levels.

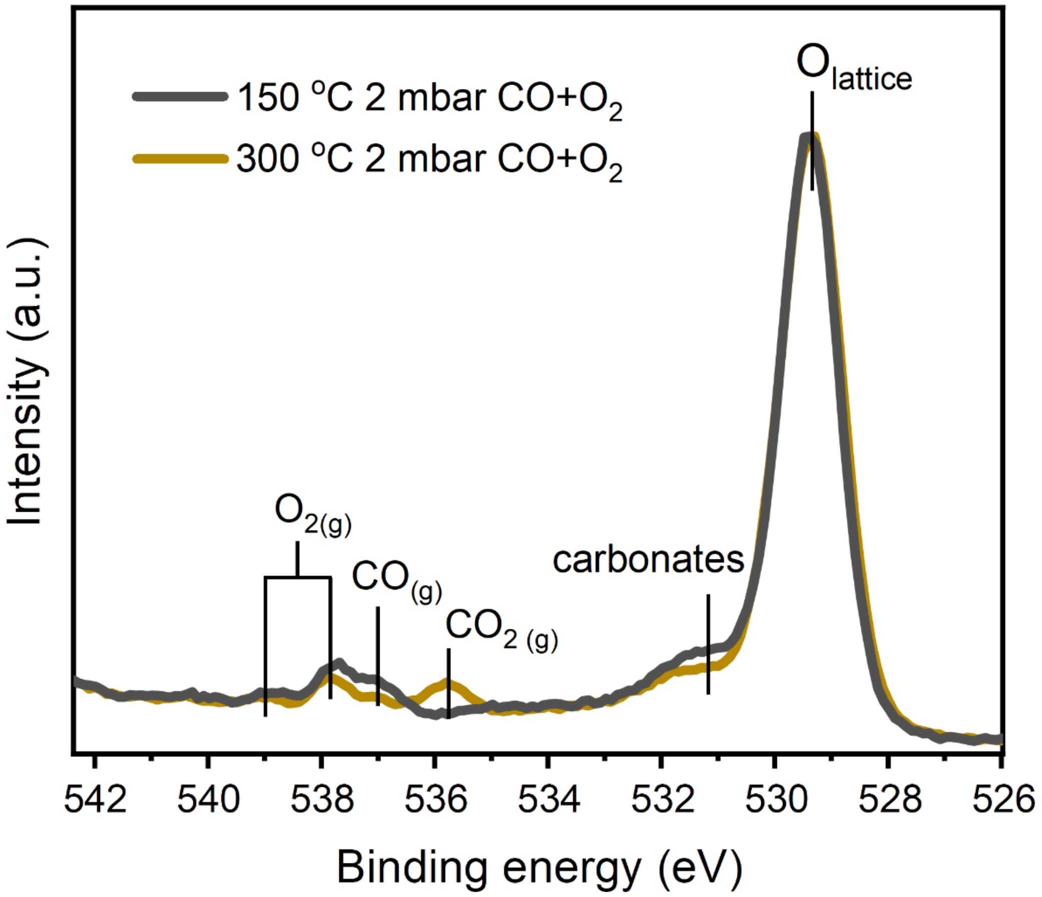

Figure S20. In situ NAP-XPS spectra of O 1s core-line acquired during CO oxidation ($CO+O_2$ =1+1 mbar) at 150 ºC and 300 ºC. As marked on the graph, at 300 ºC the CO conversion is nearly 100% in the NAP-cell. Lower conversion as compared to XAS or DRIFTS experiments is due to the lower loading of the catalyst as practically a few mg of the catalyst were drop casted on the sample holder. Another reason is the possible bypassing of the gas-phase reactants without a contact with the sample inside the NAP-cell.

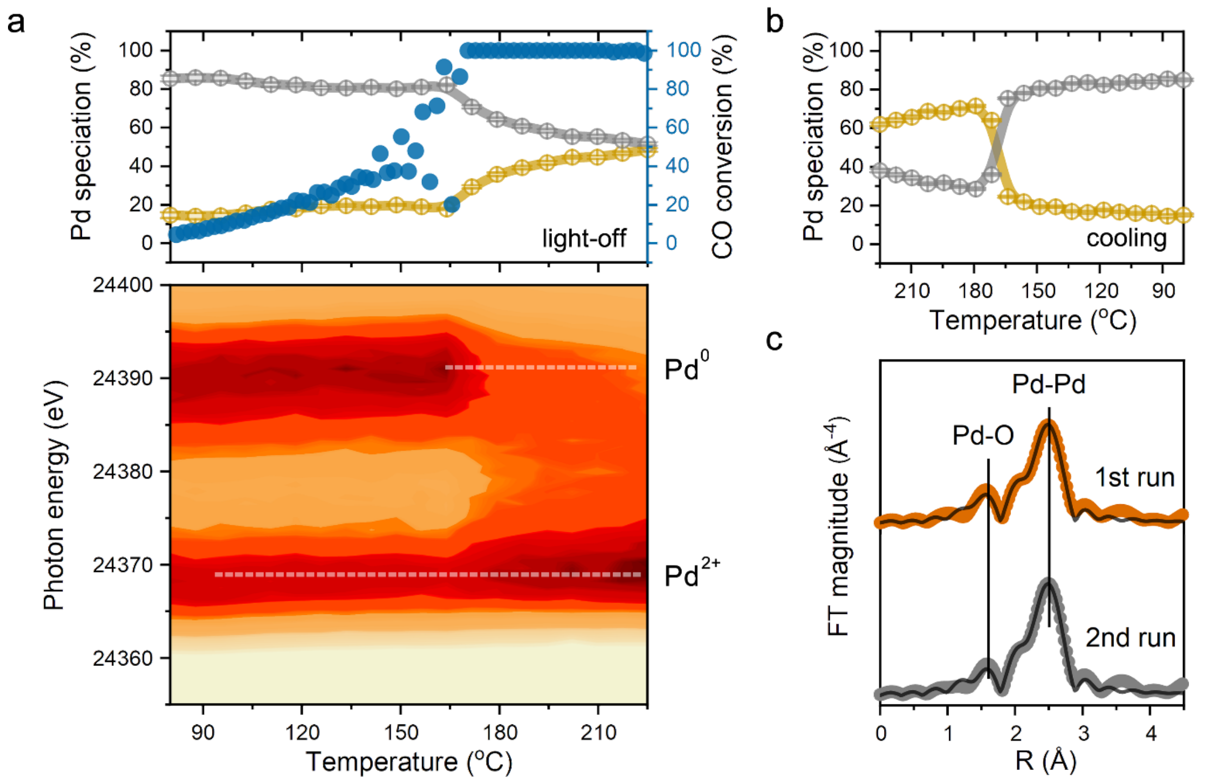

Figure S21. Evolution of Pd during CO oxidation as a function of temperature . **a,** Operando XAS at Pd K-edge during the second light-off in 1% CO and 1% O_2 in He (total flow 100 mL min^{-1}). XAS data acquired in the middle of the catalyst bed. Top panel shows the distribution of Pd states (grey-metallic, amber-oxidic), derived from linear combination fitting, and the CO conversion as a function of temperature. Bottom panel displays XANES region of Pd K-edge spectra as a function of temperature. Similar to the first light-off experiment, the concentration of metallic Pd drops when the catalyst reaches full conversion, supporting the idea of Pd reoxidation by the excess oxygen present in the 1 to 1 reaction mixture. **b**, Evolution of Pd oxidation state (grey-metallic, amber-oxidic) upon cooling in reaction mixture after second light-off test. Upon cooling, once the CO conversion decreases and the whole catalyst bed gets exposed to CO, the reduction of Pd-O species takes place, similarly to the first experiment. **c**, R-space EXAFS spectra in the middle of catalyst bed cooled to 80 ºC after the reaction. EXAFS data (Table S1) suggest that further sintering of Pd, manifested by a slight increase in Pd-Pd coordination numbers, takes place during consecutive light-off runs. Error bars in LCF analysis correspond to respective errors of the fit (Fig. S29-S30).

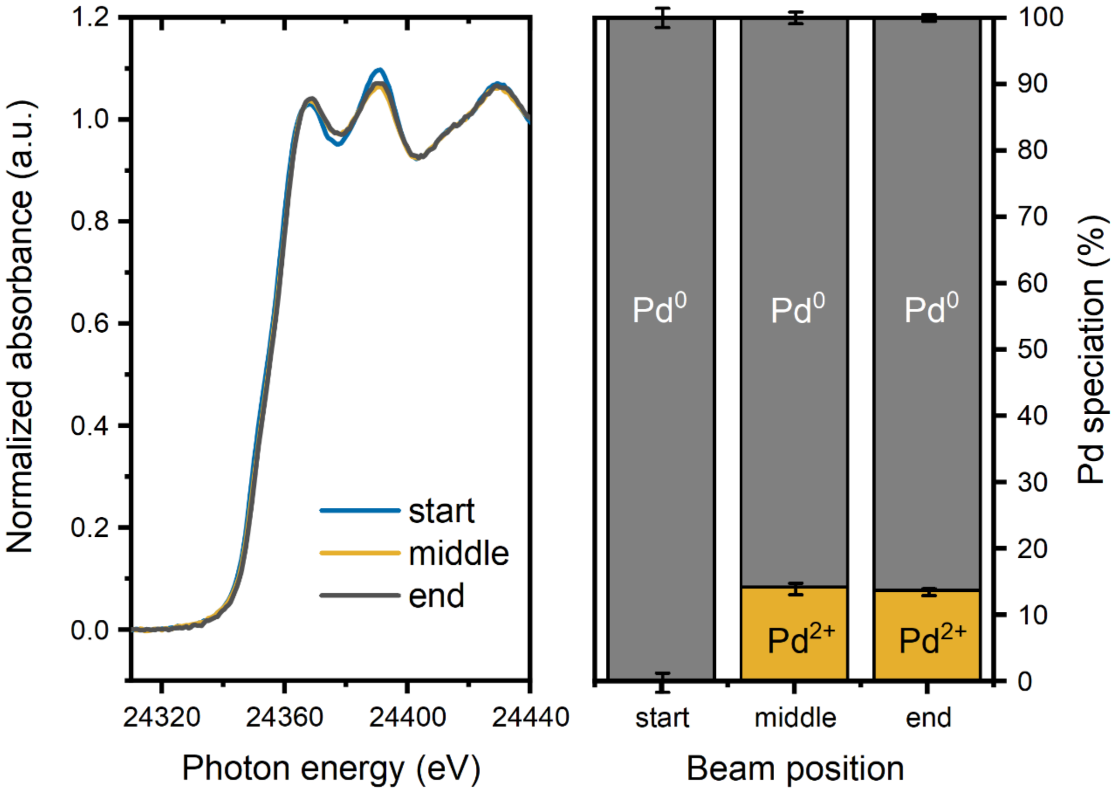

Figure S22. Operando XANES spectra at Pd K-edge acquired for Pd/CeO_2 sample during CO oxidation at 80 ºC after the second light-off cycle (80 to 225 ºC). Spectra were measured in different parts of the catalyst bed. The Pd state in the beginning of the catalyst bed is metallic whereas other parts contain oxidized Pd species.

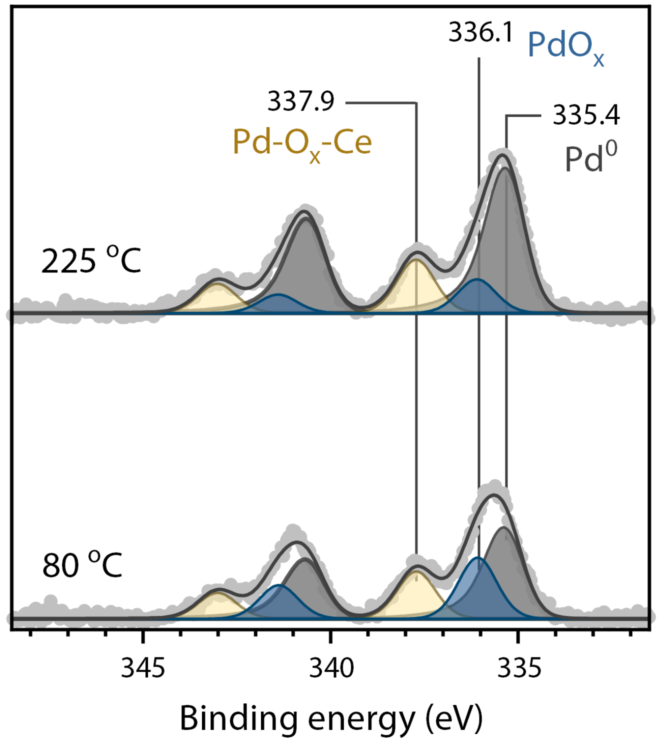

Figure S23. In situ NAP-XPS of Pd 3d core-level for the catalyst heated to 225 ºC and cooled to 80 ºC after the experiments shown in Fig. 5d. Further reduction and sintering of Pd takes place upon repeated exposure of the catalyst to reaction mixture at elevated temperatures in qualitative agreement with XAS data. The total pressure in NAP-cell was fixed to 2 mbar, for the reaction mixture 1 mbar CO and 1 mbar of O_2 was used.

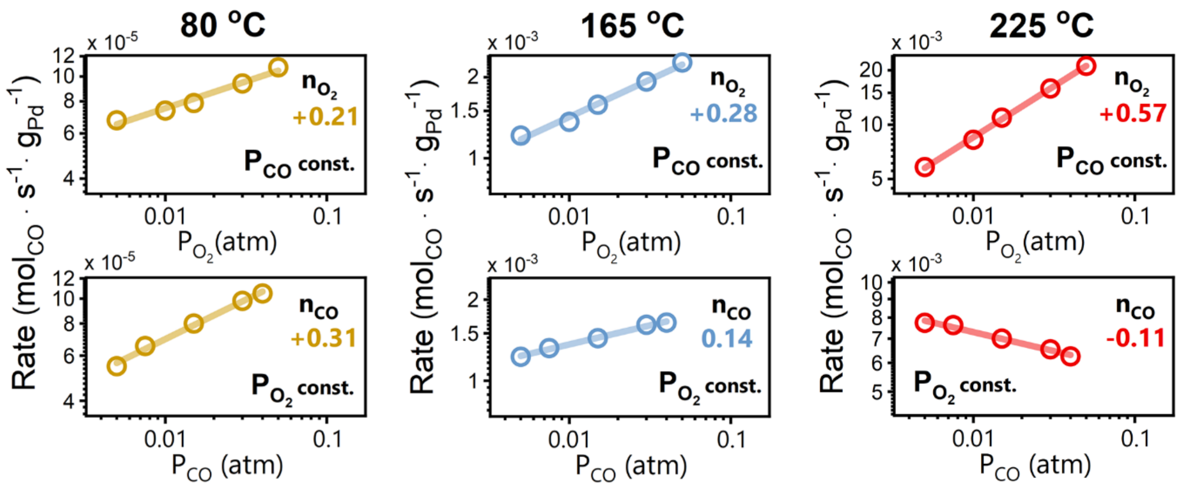

Figure S24. Reaction orders measured for Pd/CeO_2 catalyst as a function of temperature. For each measurement a fresh catalyst was loaded and stabilized in the reaction flow for at least 14 h to reach steady state. Total flow was fixed to 200 ml/min, the loading of catalysts was varied to maintain low level of CO conversion (<5%). Sieved fraction (125 to 250 μm) was diluted with ~250-300 mg of SiC. The catalyst was pretreated in 20% O_2/He at 300 ºC for 1 h.

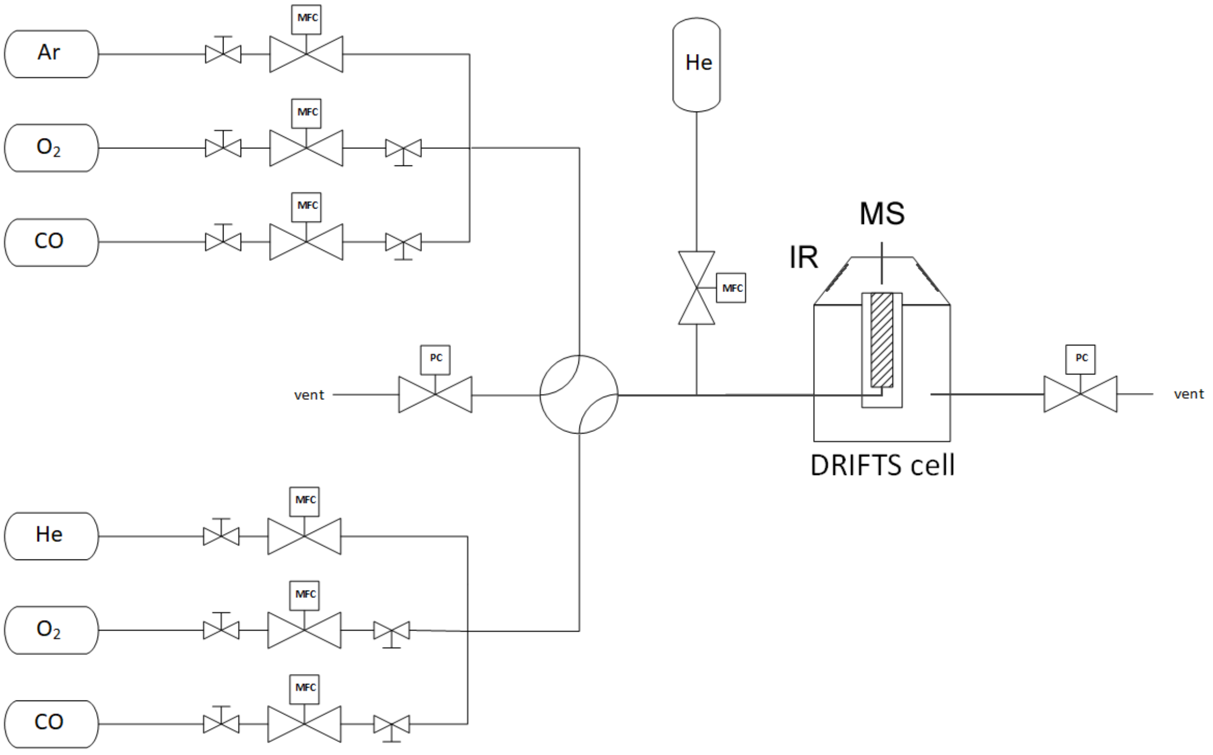

Figure S25. Scheme of the operando DRIFTS setup.

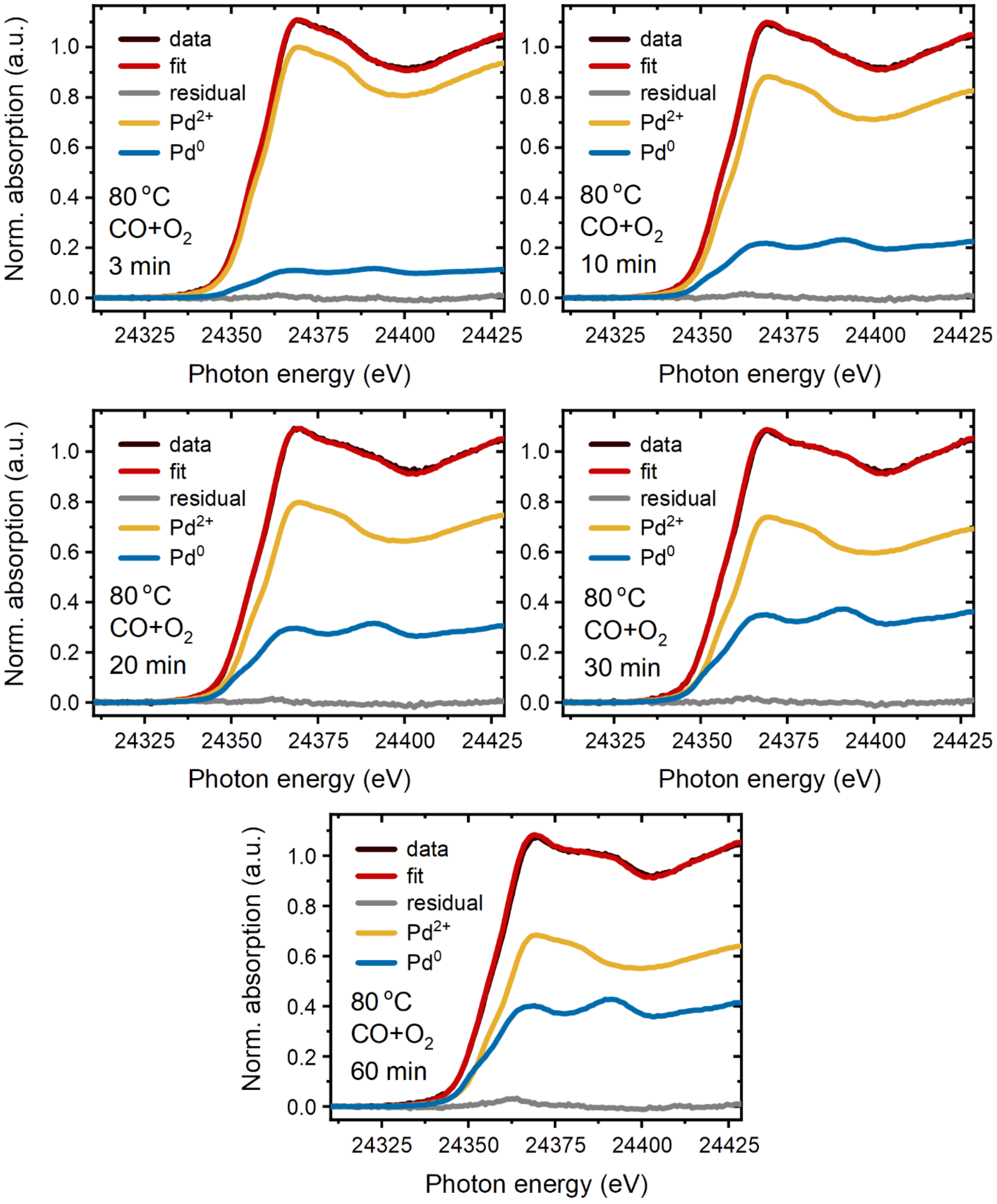

Figure S26. Results of LCF analysis of operando XAS data acquired during the experiments shown in Fig. 2a (switch from O_2 to $CO+O_2$ at 80 °C).

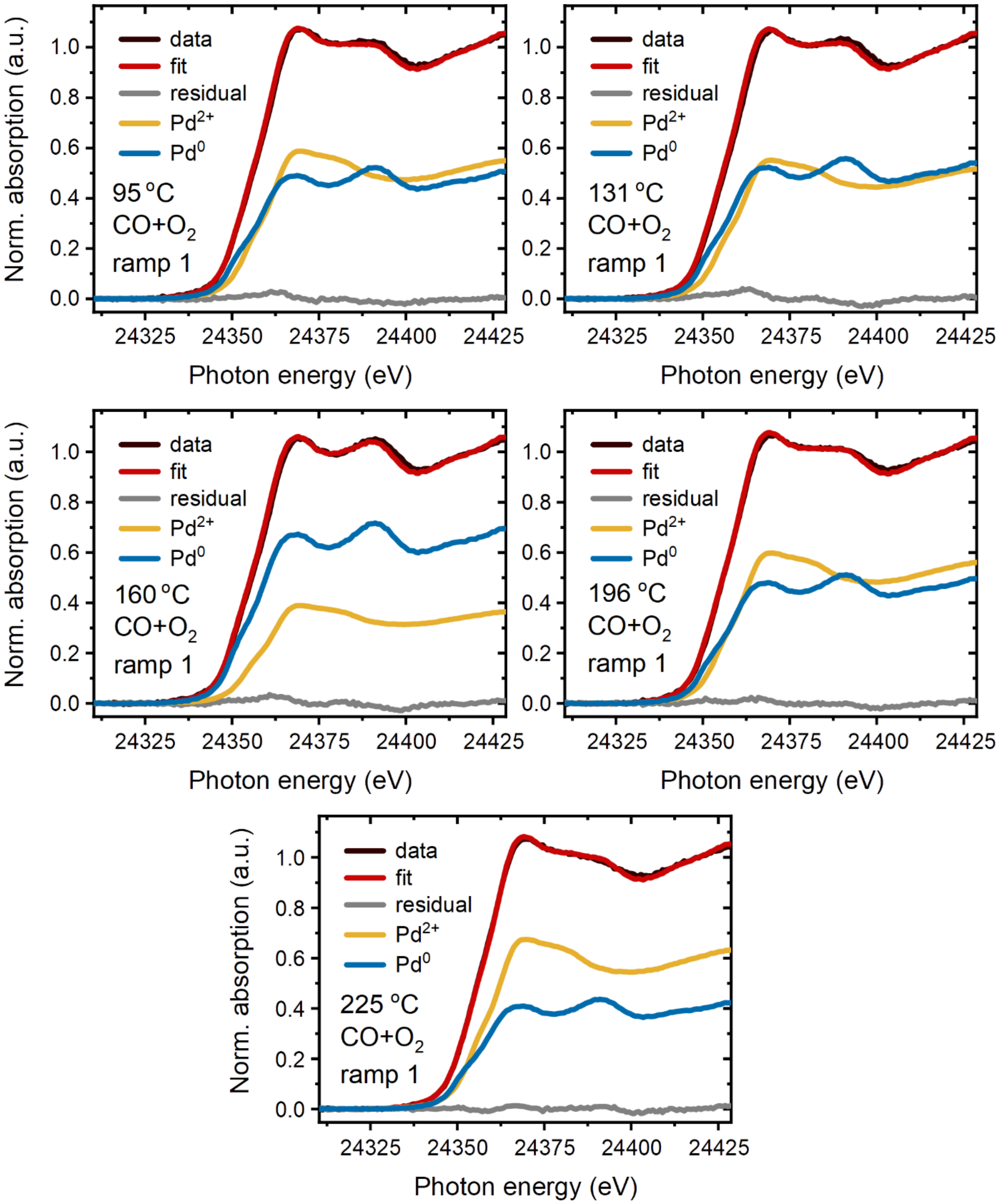

Figure S27. Results of LCF analysis of operando XAS data acquired during the experiments shown in Fig. 5a (first light-off test from 80 °C to 225 °C).

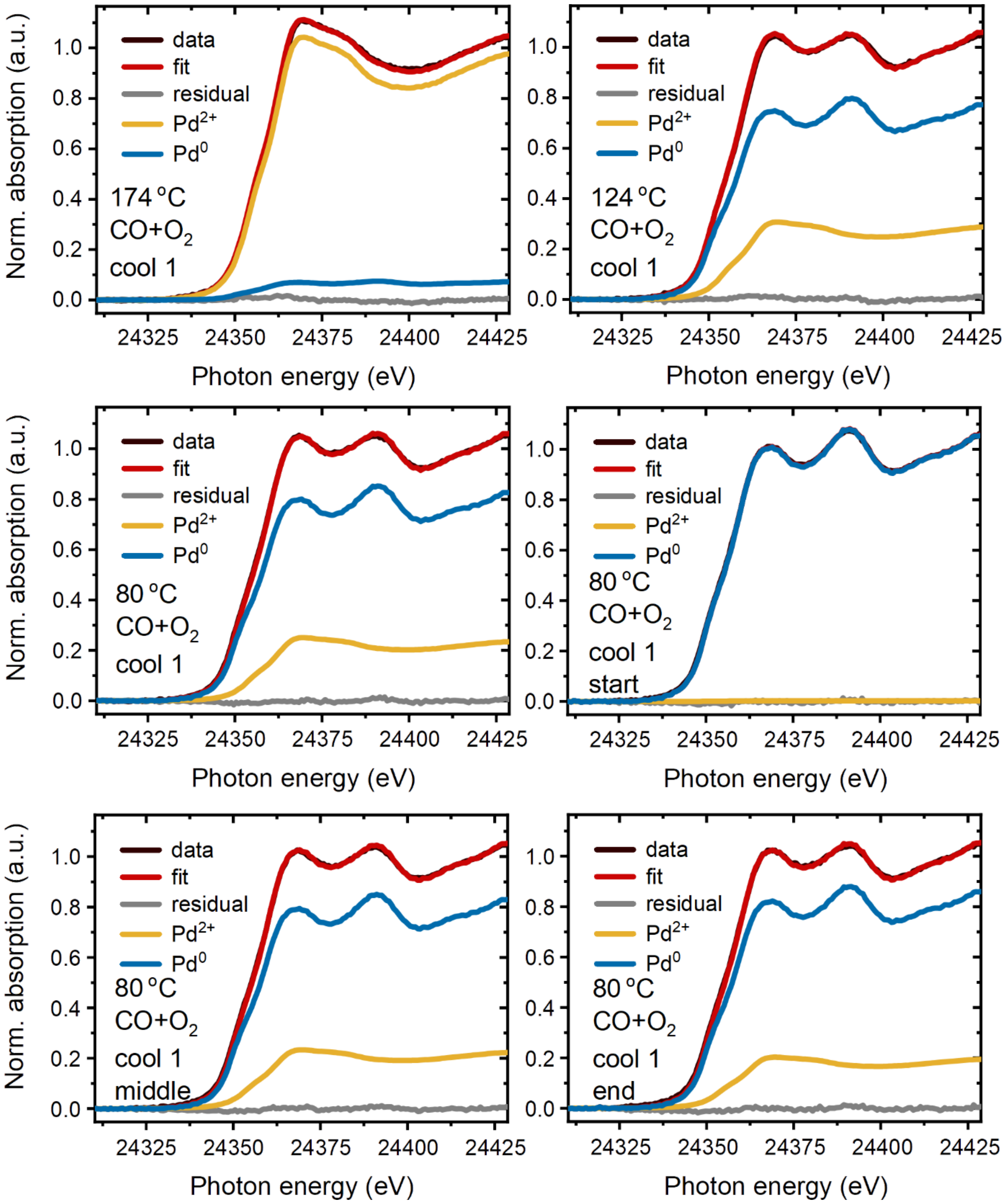

Figure S28. Results of LCF analysis of operando XAS data acquired during the experiments shown in Fig. 5b,c (cooling from 225 °C to 80 °C in reaction mixture and probing different parts of the catalyst bed).

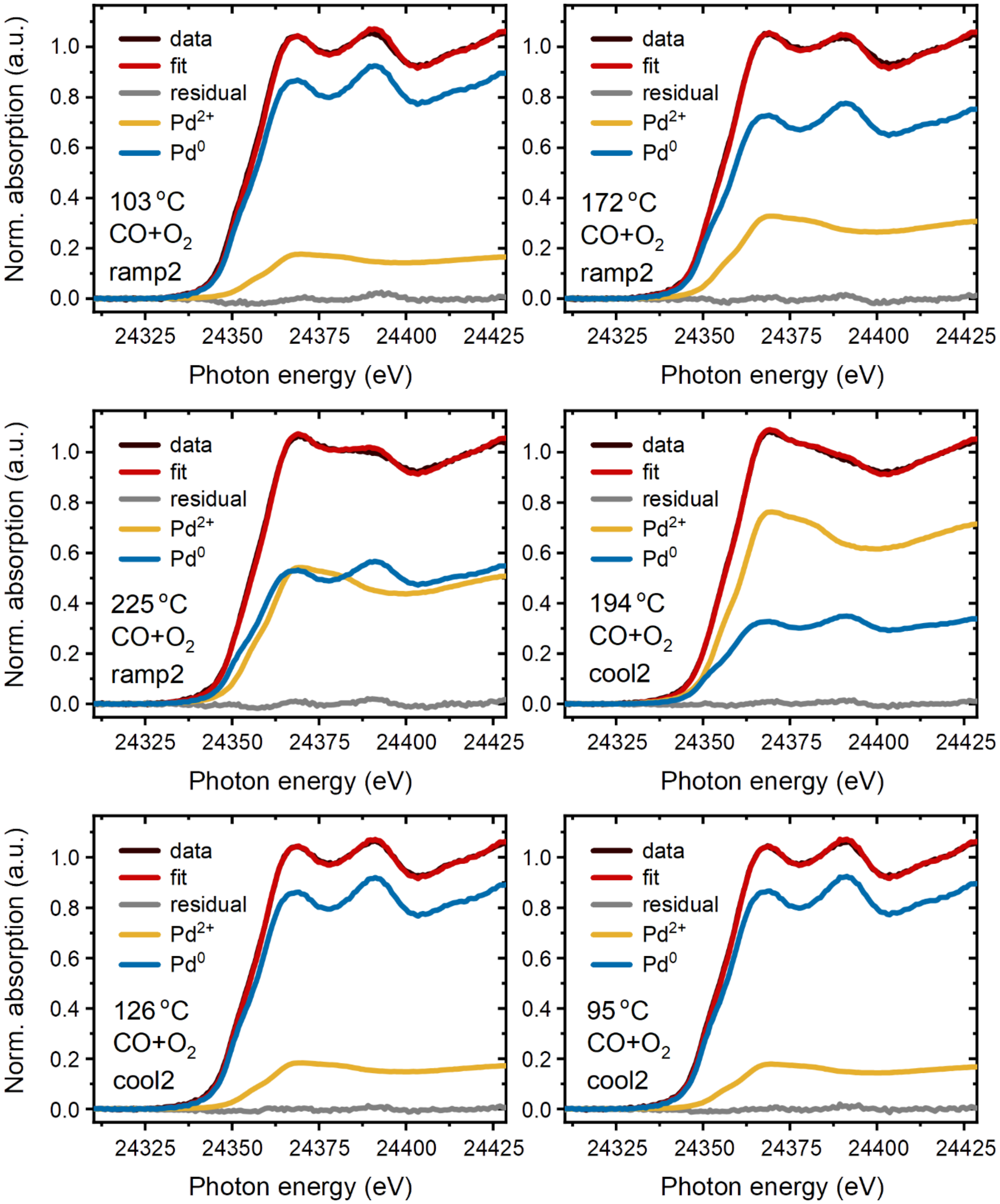

Figure S29. Results of LCF analysis of operando XAS data acquired during the experiments shown in Fig. S21a,b (second light-off from 80 °C to 225 °C with subsequent cooling to 80 °C in reaction mixture).

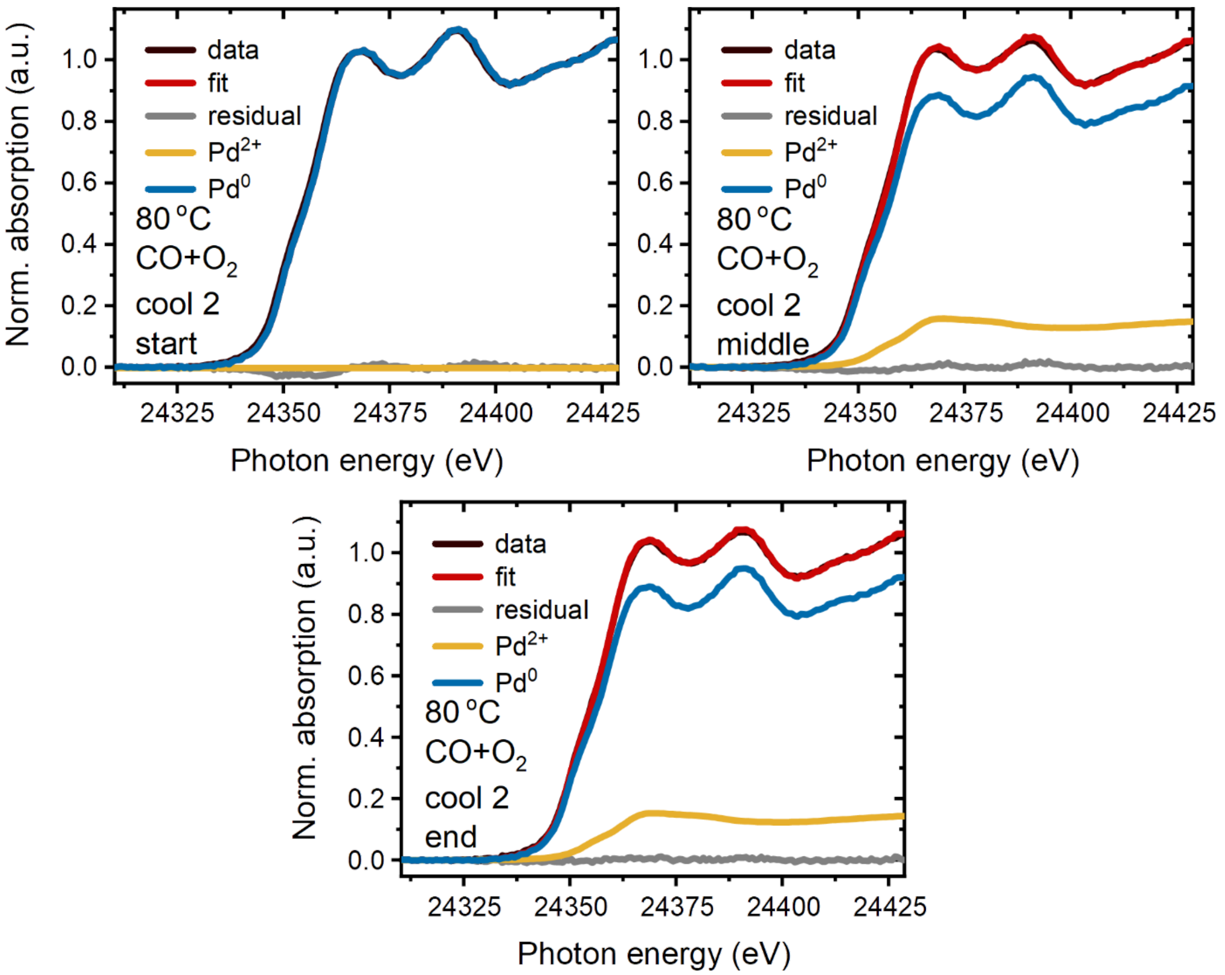

Figure S30. Results of LCF analysis of operando XAS data acquired in different parts of the catalyst bed after second light-off test.

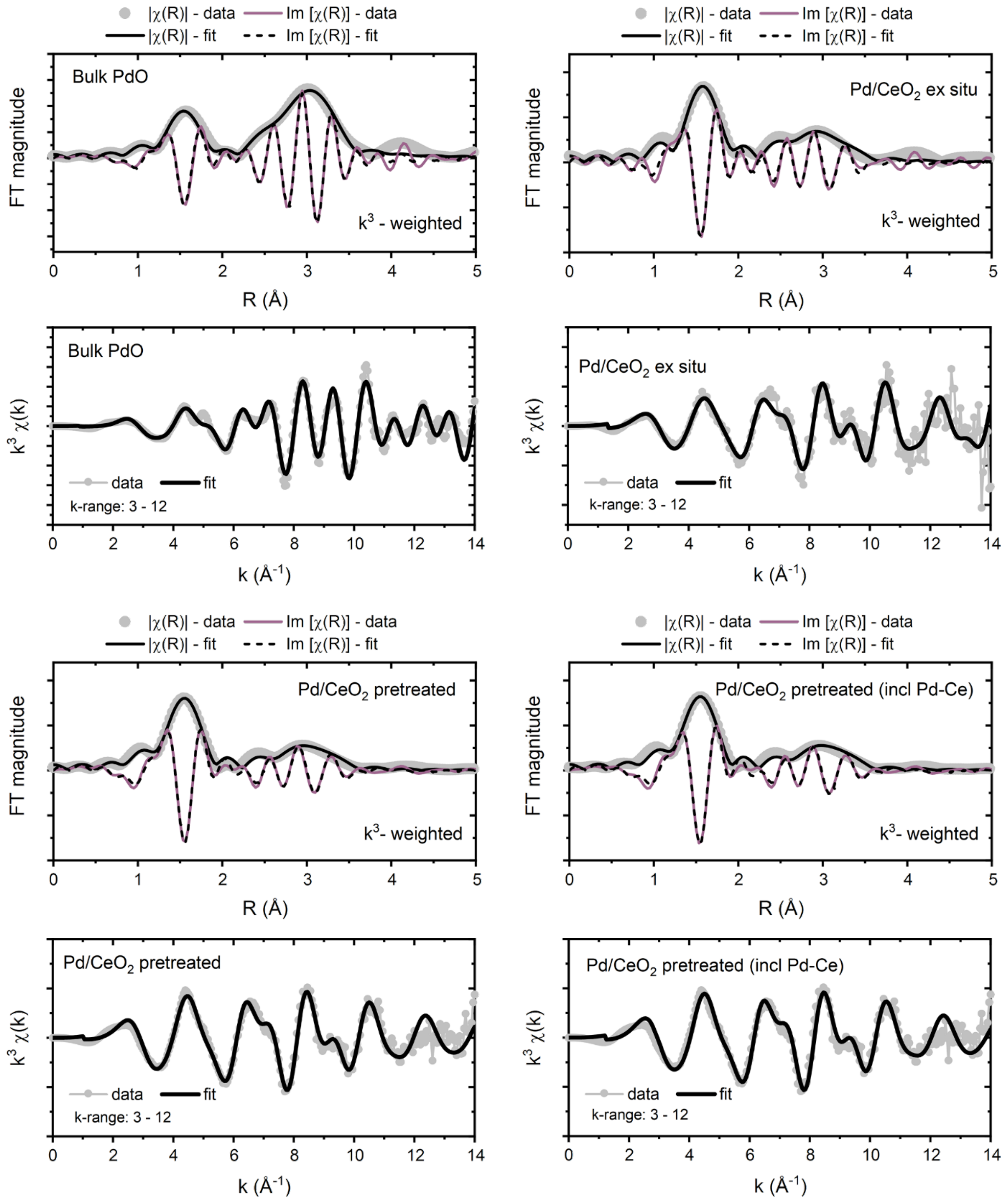

Figure S31. Results of the EXAFS data fitting shown in R and k space. Details are listed in Table S1.

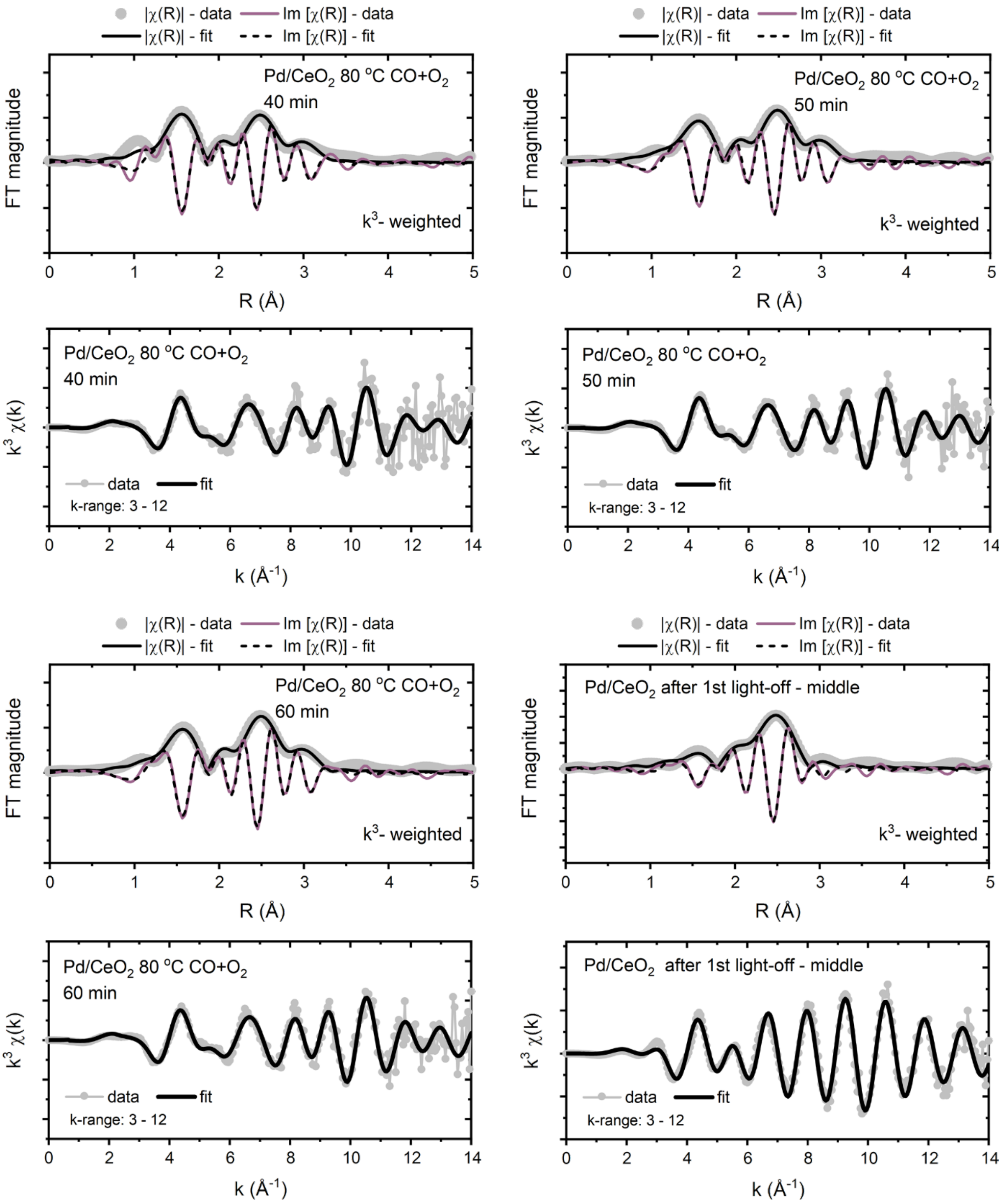

Figure S32. Results of the EXAFS data fitting shown in R and k space. Details are listed in Table S1.

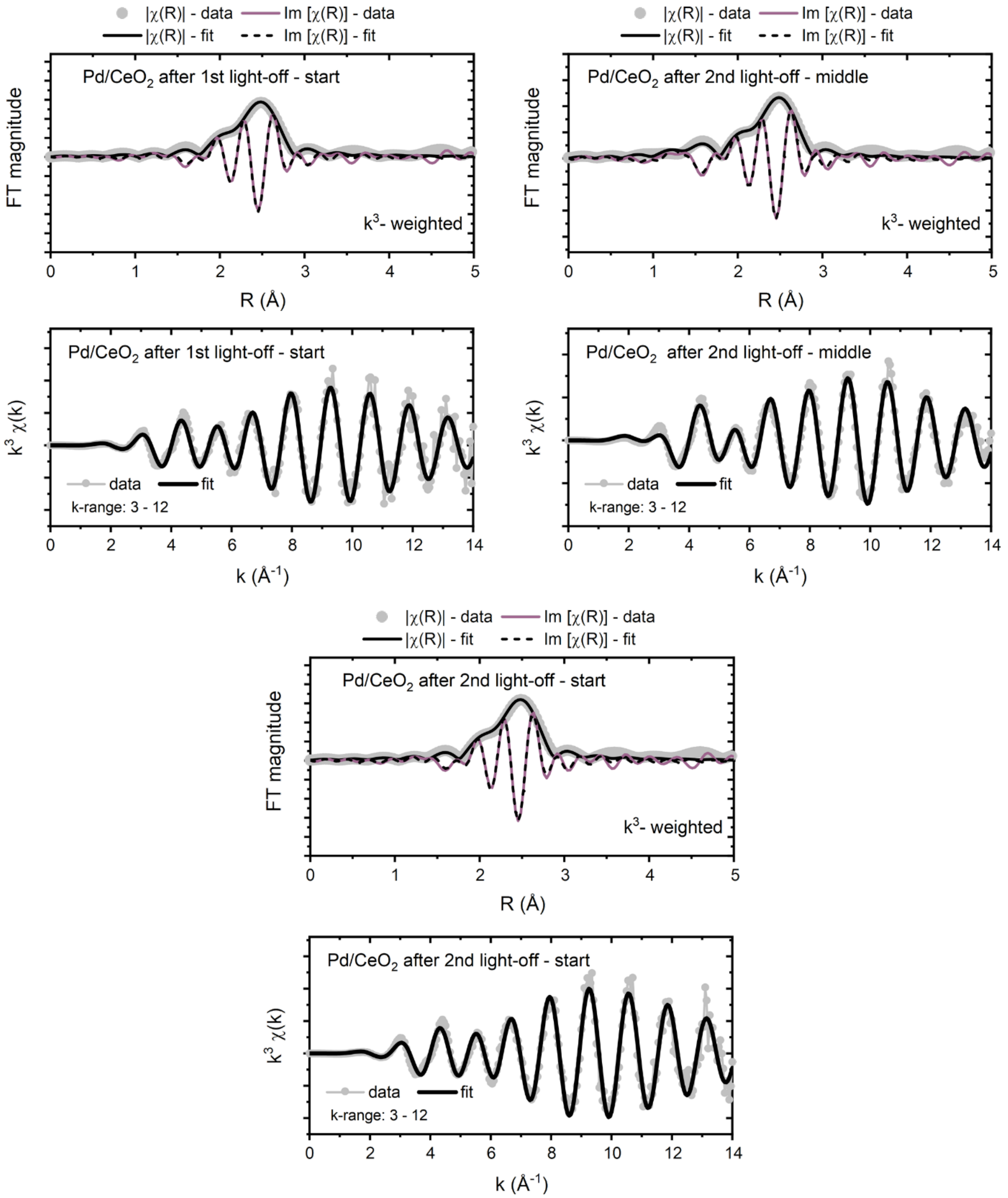

Figure S33. Results of the EXAFS data fitting shown in R and k space. Details are listed in Table S1.

Table S1. EXAFS fitting results.

Sample	Path	R [Å]	CN	σ^2 [Å^2]	ΔE_0 [eV]	R-factor [%]
**Pd/CeO_2 ex situ**	Pd-O	2.02 ± 0.01	3.8 ± 0.4	0.001 ± 0.001	6.5 ± 1.2	1.7
	Pd-Pd (oxide)	3.02 ± 0.01	1.6 ± 1.1	0.004 ± 0.004		
	Pd-Pd (oxide)	3.42 ± 0.01	2.3 ± 1.5	0.004 ± 0.004		
**Pd/CeO_2 O_2 80 ºC**	Pd-O	2.01 ± 0.01	4.1 ± 0.3	0.003 ± 0.001	4.1 ± 0.9	1.0
	Pd-Pd (oxide)	3.01 ± 0.01	2.7 ± 1.3	0.011 ± 0.003		
	Pd-Pd (oxide)	3.41 ± 0.01	5.1 ± 2.2	0.011 ± 0.003		
**Pd/CeO_2 O_2 80 ºC (incl. Pd-Ce scattering)**	Pd-O	2.01 ± 0.01	4.2 ± 0.3	0.003 ± 0.001	5.5 ± 1.0	1.1
	Pd-Pd (oxide)	3.01 ± 0.01	2.4 ± 1.8	0.012 ± 0.004		
	Pd-Pd (oxide)	3.40 ± 0.01	5.5 ± 2.8	0.012 ± 0.004		
	Pd-Ce	3.27 ± 0.01	0.5 ± 1.0	0.012 ± 0.004		
**Pd/CeO_2 $CO+O_2$ 80 ºC – 40 min**	Pd-O	2.01 ± 0.02	2.5 ± 0.5	0.002 ± 0.002	1.8 ± 2.0	2.0
	Pd-Pd (metal)	2.78 ± 0.02	5.2 ± 1.6	0.010 ± 0.002		
	Pd-Pd (oxide)	3.01 ± 0.02	4.2 ± 2.1	0.010 ± 0.002		
	Pd-Pd (oxide)	3.41 ± 0.02	1.8 ± 1.5	0.010 ± 0.002		
**Pd/CeO_2 $CO+O_2$ 80 ºC – 50 min**	Pd-O	2.00 ± 0.01	2.6 ± 0.4	0.004 ± 0.002	1.2 ± 1.6	1.2
	Pd-Pd (metal)	2.77 ± 0.01	5.5 ± 1.2	0.010 ± 0.002		
	Pd-Pd (oxide)	3.10 ± 0.02	4.0 ± 1.6	0.010 ± 0.002		
	Pd-Pd (oxide)	3.40 ± 0.02	1.7 ± 1.1	0.010 ± 0.002		
**Pd/CeO_2 $CO+O_2$ 80 ºC – 60 min**	Pd-O	2.01 ± 0.02	2.6 ± 0.5	0.004 ± 0.002	1.9 ± 1.7	1.5
	Pd-Pd (metal)	2.78 ± 0.01	5.7 ± 1.4	0.010 ± 0.002		
	Pd-Pd (oxide)	3.01 ± 0.02	4.3 ± 1.8	0.010 ± 0.002		
	Pd-Pd (oxide)	3.41 ± 0.02	1.5 ± 1.2	0.010 ± 0.002		

**Pd/CeO$_2$ CO+O$_2$ 80 ºC after 1st light-off - middle**	Pd-O	1.97 ± 0.02	1.6 ± 0.7	0.006 ± 0.006	-2.6 ± 0.9	1.3
	Pd-Pd (metal)	2.74 ± 0.01	6.9 ± 0.9	0.008 ± 0.001		
**Pd/CeO$_2$ CO+O$_2$ 80 ºC after 1st light-off - inlet**	Pd-Pd (metal)	2.74 ± 0.01	9.6 ± 1.0	0.007 ± 0.001	-3.0 ± 0.7	1.0
**Pd/CeO$_2$ CO+O$_2$ 80 ºC after 2nd light-off - middle**	Pd-O	1.97 ± 0.02	1.7 ± 0.8	0.008 ± 0.007	-2.3 ± 0.8	1.0
	Pd-Pd (metal)	2.74 ± 0.01	7.3 ± 0.9	0.007 ± 0.001		
**Pd/CeO$_2$ CO+O$_2$ 80 ºC after 2nd light-off - inlet**	Pd-Pd (metal)	2.74 ± 0.01	9.7 ± 0.9	0.007 ± 0.001	-1.6 ± 0.7	1.0
**PdO**	Pd-O	2.00 ± 0.01	4.0	0.002 ± 0.001	2.9 ± 1.3	2.1
	Pd-Pd (oxide)	3.04 ± 0.01	4.0	0.004 ± 0.001		
	Pd-Pd (oxide)	3.43 ± 0.01	8.0	0.004 ± 0.001		
**Pd foil**	Pd-Pd	2.74 ± 0.01	12.0	0.006 ± 0.001	-0.1 ± 0.5	0.3

S_0^2 values (0.68 for B18, Diamond and 0.82 for P65, DESY) were derived from fitting the PdO reference (Sigma-Aldrich) and used for the fitting of Pd-CeO$_2$ samples. ΔE_0 was shared for all the shells, while σ^2 and ΔR values were shared within each shell to obtain stable fit and obey Nyquist criterion. The errors indicate the uncertainties of fitting results obtained from Artemis software.

Table S2. XPS data quantification.

	Pd/Ce(at. ratio)	Pd^0 (%)	PdO_x (%)	Pd^{2+}-O-Ce (%)
**80 ºC O_2**	0.044	0	13	87
**80 ºC $CO+O_2$**	0.047	5	35	60
**150 ºC $CO+O_2$**	0.041	47	7	46
**225 ºC $CO+O_2$**	0.039	53	6	40
**300 ºC $CO+O_2$**	0.028	53	5	42
**80 ºC $CO+O_2$ cool**	0.028	56	11	33

Table S3. Basic characterization.

	Pd loading (wt%, ICP)	BET, m^2g^{-1}
**Pd/CeO_2**	0.93 ± 0.02	39.8
**Pd/ZrO_2**	0.72 ± 0.01	6.2

References

(1) Stadnichenko, A. I.; Muravev, V. V.; Koscheev, S. V.; Zaikovskii, V. I.; Aleksandrov, H. A.; Neyman, K. M.; Boronin, A. I., *Surf. Sci.* **2019**, *679*.

(2) Kato, S.; Ammann, M.; Huthwelker, T.; Paun, C.; Lampimäki, M.; Lee, M.-T.; Rothensteiner, M.; van Bokhoven, J. A., *Phys. Chem. Chem. Phys.* **2015**, *17* (7), 5078–5083.

(3) Parastaev, A.; Muravev, V.; Huertas Osta, E.; van Hoof, A. J. F.; Kimpel, T. F.; Kosinov, N.; Hensen, E. J. M., *Nat. Catal.* **2020**, *3*, 526–533.

(4) Skála, T.; Šutara, F.; Prince, K. C.; Matolín, V., *J. Electron Spectrosc. Relat. Phenom.* **2009**, *169* (1), 20–25.

(5) Soubaihi, R. M.; al Saoud, K. M. & Dutta, J., *Catalysts* **2018**, *8,* 660 (2018).
